# Ca_v_2.2-NFAT2-USP43 axis promotes invadopodia formation and breast cancer metastasis through cortactin stabilization

**DOI:** 10.1038/s41419-022-05174-0

**Published:** 2022-09-22

**Authors:** Ying Xue, Min Li, Jie Hu, Yuanlin Song, Wei Guo, Changhong Miao, Di Ge, Yingyong Hou, Xuefei Wang, Xingxu Huang, Tianshu Liu, Xiaoping Zhang, Qihong Huang

**Affiliations:** 1 Cancer Center, Zhongshan Hospital, Fudan University, Shanghai, PR China; 2 Institute of Clinical Sciences, Zhongshan Hospital, Fudan University, Shanghai, PR China; 3 Department of Pulmonary and Critical Care Medicine, Zhongshan Hospital, Fudan University, Shanghai, PR China; 4 Department of Laboratory Medicine, Zhongshan Hospital, Fudan University, Shanghai, PR China; 5 Department of Anesthesiology, Zhongshan Hospital, Fudan University, Shanghai, PR China; 6 Department of Thoracic Surgery, Zhongshan Hospital, Fudan University, Shanghai, PR China; 7 Department of Pathology, Zhongshan Hospital, Fudan University, Shanghai, PR China; 8 Department of General Surgery/Gastric Cancer Center, Zhongshan Hospital, Fudan University, Shanghai, PR China; 9 School of Life Science and Technology, ShanghaiTech University, Shanghai, PR China; 10 Department of Medicial Oncology, Zhongshan Hospital, Fudan University, Shanghai, PR China; 11 The Institute of Intervention Vessel, Tongji University School of Medicine, Shanghai, PR China; 12 Shanghai Respiratory Research Institute, Shanghai, PR China

**Keywords:** Invadopodia, Actin

## Abstract

Distant metastasis is the main cause of mortality in breast cancer patients. Using the breast cancer genomic data from The Cancer Genome Atlas (TCGA), we identified brain specific Ca_v_2.2 as a critical regulator of metastasis. Ca_v_2.2 expression is significantly upregulated in breast cancer and its higher expression is inversely correlated with survival suggesting a previously unappreciated role of Ca_v_2.2 in breast cancer. Ca_v_2.2 is required for breast cancer migration, invasion, and metastasis. Interestingly, Ca_v_2.2 promotes invadopodia formation and extracellular matrix (ECM) degradation through the stabilization of invadopodia component cortactin in a proteosome-dependent manner. Moreover, deubiquitinating enzyme USP43 mediated the functions of Ca_v_2.2 in cortactin stabilization, invadopodia formation, ECM degradation, and metastasis. Interestingly, Ca_v_2.2 upregulates USP43 expression through NFAT2 dephosphorylation and nuclear localization. Our study uncovered a novel pathway that regulates cortactin expression and invadopodia formation in breast cancer metastasis.

## Introduction

Distant metastasis is the main cause of mortality in breast cancer patients [1]. Patients with metastatic breast cancer generally presents poor prognosis, with an average 5-year survival rate of about 27% [1]. Although surgery, targeted therapy, and radiation therapy have been used to control primary tumor growth, they are not very effective in preventing relapses and in the management of breast cancer metastases. Identification of novel regulators of metastasis may provide potential prognostic markers and therapeutic targets for cancer intervention.

Ca_v_2.2 belongs to a voltage-dependent calcium channel family, which is required for maintaining Ca^2+^ homeostasis in normal cells [2]. Ca_v_2.2 is expressed in excitable cells such as neurons, allowing calcium influx after responding to a depolarization of membrane potential [3]. It regulates SNARE-mediated release of neurotransmitters, eventually resulting in synaptic transmission [4]. It is also the main force in supporting transmission of sensory information from nociceptors to spinal cord dorsal horn neurons. Specific inhibition of Ca_v_2.2 such as ziconotide, an FDA-approved drug, can be used for chronic pain [5–8]. Modulation of Ca_v_2.2 is involved in synaptic plasticity, synaptogenesis, gene transcription, neuronal survival, and the migration of immature neurons [9, 10]. Moreover, bi-allelic loss-of-function Ca_v_2.2 variants have been found in progressive epilepsy-dyskinesia, leading to disruption of Ca^2+^ influx and subsequent impaired synaptic neurotransmission [11]. These studies demonstrated that Ca_v_2.2 plays an irreplaceable role in normal human neural development. However, whether Ca_v_2.2 is involved in cancer development is largely unknown.

Cancer cells endowed with the ability to degrade extracellular matrix (ECM) can invade surrounding tissues, a crucial step toward in the spreading of the primary tumor and metastasis formation [12, 13]. Notably, invasive cells accomplish this process through invadopodia, a special protruded cell membrane structure with proteolytic activity [14–19]. The protrusive ability of invadopodia is obtained by combining the physical force generated by actin polymerization with the chemical activity of matrix metalloproteinase (MMP)-mediated ECM degradation [20]. A number of signaling pathways including cytoskeletal modulators, adhesive molecules, membrane trafficking regulate invadopodia formation [21, 22]. Actin binding protein cortactin plays an essential role in invadopodia formation [23, 24]. It directly binds to Arp2/3 and N-WASP to promote actin assembly at invadopodia puncta [25–30]. In addition, cortactin regulates ECM degradation through facilitating the secretion of matrix metalloproteinases [24, 27, 31]. It has been shown that cortactin can be regulated by phosphorylation and acetylation [32–36]. However, it has not been studied whether deubiquitination process can regulate cortactin and its functions in invadopodia formation.

Using bioinformatic analysis of TGCA data of breast cancer, we discovered that high Ca_v_2.2 expression was inversely correlated with breast cancer overall survival, especially in triple negative breast cancer patients. We showed that Ca_v_2.2 expression was upregulated in breast cancer tissues and cell lines but was absent in normal breast epithelial cells as well as normal breast tissues. We demonstrated that Ca_v_2.2 was required for breast cancer migration, invasion, and metastasis. Mechanistically we showed that Ca_v_2.2 promoted invadopodia formation and focal matrix degradation. Conversely, knockdown of Ca_v_2.2 expression suppressed invadopodia formation and ECM degradation. In addition, we showed that deubiquitinating enzyme USP43 stabilized cortactin and mediated the functions of Ca_v_2.2 in invadopodia formation and metastasis in breast cancer. Taken together, we discovered a novel pathway that Ca_v_2.2 regulated invadopodia formation through deubiquitination process.

## Materials and methods

### Cell culture, plasmids and antibodies

MDA-MB-231, MDA-MB-436, BT-549 were purchased from Cell Bank of the Chinese Academy of Sciences (Shanghai, China). All cell lines were maintained at 37 °C in RPMI 1640 medium supplemented with 10% fetal bovine serum (FBS), 100 IU/ml penicillin and 100 μg/ml streptomycin.

Ca_v_2.2 plasmid was purchased from Addgene (Plasmid: #62574). Full length of USP43 and Cortactin were amplified by PCR and cloned into Flag-HA-Phase vector and Flag-pcDNA3.1 vector. Full length of NFAT2 was amplified by PCR and cloned into PCDH vector.

Anti-Ca_v_2.2 was purchased from Proteintech, and antibodies against Cortactin, USP43, NFAT2, p-NFAT2 were purchased from Abcam. Anti-ubiquitin was purchased from Santa Cruze. Antibodies against Flag, IgG, NFAT3 were purchased from Cell signaling.

### Transfection and virus production

For transient transfection, cells were transfected with Lipofectamine 2000 according to manufacturer’s procedure. Briefly, cells were seeded 24 h prior to transfection. The plasmid(s) of interest and Lipofectamine reagent were mixed in Opti-MEM media. Media was then replaced with regular media without antibiotics 6 h post-transfection. Control shRNA, Ca_v_2.2 shRNA, USP43 shRNA, NFAT2 shRNA, cortactin cDNA or vector along with the packaging plasmids PMD2.G and PSPAX were transfected using Lipofectamine 2000 (Invitrogen) to produce lentivirus as previously described [37]. Infectious lentiviruses were collected 24 h and 48 h after transfection, centrifuged to remove cell debris and filtered through 0.22 mm filters (Millipore).

### CCK8 (Cell counting kit) assay

Cells were seeded in 96-well plates for 24 hours (h), 48 h, 72 h, 96 h. Culture medium was removed, then fresh medium was added to each well along with 10 μl of CKK8 solution (Takara). The absorbance was measured at a wavelength of 450 nm after incubation at 37 °C for 4 h.

### Cell counting assay

Cell number was determined using a Hemocytometer. Briefly, 50000 cells were seeded in 12-well plates for 24 h, 48 h, 72 h, 96 h, washed with PBS, digested with trypsin followed by adding supplemented DMEM and mixing cells by pipetting until cells formed a single cell suspension. Hemocytometer was used to determine cell number.

### Transwell migration and invasion assay

In vitro cell migration and invasion assays were performed as described previously [38]. Briefly, Cells were incubated in serum-free medium for 24 h, and then were collected, counted, suspended in serum-free medium. Cell suspension were seeded in the upper chamber containing 200 ml serum-free medium with an uncoated or Matrigel-coated membrane, while 600 ml RPMI1640 medium with 10% FBS was added into the lower chamber. After cultured for 48 h, cells were fixed using 4% methyl alcohol for 15 min following staining via 5% crystal violet solution for additional 15 min. Five fields of view were randomly selected and photographed using an upright Metallurgical Microscope (Olympus, Japan).

### Quantification of Ca_v_2.2 expression in human tissues

Total RNAs of normal human tissues were purchased from Clontech and cDNA was synthesized from total RNA using Reverse Transcription Kit (Takara) according to the manufacturer’s instructions. Quantitative real-time PCR was carried out by QuantiTect SYBR Green PCR Kit (Takara). The average of three independent analyses was used.

### Quantitative real-time PCR

Total RNAs were extracted from cells at utilizing Trizol reagent (Takara) and reverse transcribed with cDNA Reverse Transcription Kit (Takara) according to the manufacturer’s instructions. Quantitative real-time PCR was performed by QuantiTect SYBR Green PCR Kit (Takara) using an Applied Biosystems Q5 Real-time PCR system. Relative mRNA expressions were determined by 2−delta delta (Ct-Cc) where Ct and Cc are the mean threshold cycle differences after normalizing to beta-actin values.

### Tumor transplantation in mice

In vivo tumor transplantation assay was performed as described previously [38]. The MDA-MB-436 human breast cancer cell lines stably expressing Firelfly Luciferase gene with Ca_v_2.2 shRNA or control shRNA were suspended in 100 μl of PBS and injected in the lateral tail vein of 6–8 weeks old female NOD/SCID mice. Then, IVIS 200 Imaging system (Xenogen Corporation, Hopkinton, MA) was used to image mice bearing luciferase positive tumors. The primary tumors and lung metastasis were analyzed using bioluminescent flux (photons per second per steradian per centimeter square). In order to minimize the number of animals we used power analysis to calculate the minimum sample size using the free software DOEUMH (https://samplesizeumh.shinyapps.io/DOEUMH) based on the TrialSize library of the R program (R Core Team). We selected the procedure KMeans – ANOVA, fixing the significance to 0.05, power to 0.08, and a drop-out of 5%. We took into consideration differences between averages of about 1.5–2 fold. Minimum number of mice/group: 5–6 mice/group. Animal experiment protocols were approved by the Institutional Animal Care and Use Committee (IACUC) of the Zhongshan Hospital. Animal procedures were conducted in compliance with the IACUC.

### Invadopodia formation assay

Sterile coverslips were incubated in 0.1 mg/ml poly-D-lysine for 10 minutes at room temperature followed by incubating 2% gelatin (Sigma) for 1 h at 37 °C. Then, coverslips were added 0.25% gluteraldehyde/PBS for 15 min followed by PBS wash three times. Coverslips were then incubated for 15 min in 5 mg/ml NaBH_4_ followed by PBS wash three times. The coverslips were then incubated at 37 °C in 10% calf serum/RPMI1640 for 2 h. Cells were seeded on each coverslip and incubated for 8 h, and fixed at 37 °C in 4% paraformaldehyde (PFA)/PBS for 15 min, permeabilized with 0.1% Triton X-100/PBS for 10 min, and blocked with 3% goat serum. Samples were incubated with primary antibodies overnight at 4 °C and with secondary antibodies and/or phalloidin for 2 h. Images were taken using Olympus BX43F microscope. Co-localization of cortactin (green) and F-actin(red) puncta was used to identify invadopodia.

### Focal ECM degradation assay

Oregon Green™ gelatin 488 (Invitrogen) was used for focal gelatin degradation assay. Briefly, sterile coverslips were coated with a mix of 0.1 mg ml^−1^ poly-d-lysine followed by incubation with 0.4% glutaraldehyde for 20 min and PBS wash three times. Oregon Green™ gelatin 488 was diluted 1:50 with 0.1% unconjugated gelatin and used to coat coverslips at 37 °C for 1 h. Coverslips were then incubated with 5 mg/ml NaBH_4_ for 1 min, followed by 70% ethanol for 20 min. Three washes with 1 × PBS were performed between each step. RPMI1640 media was added to the coverslips at 37 °C for 1 h before cell plating. The coverslips were then incubated at 37 °C in 10% calf serum/RPMI1640 for 2 h. Cells were seeded on each coverslip, incubated for 8 h, fixed at 37 °C in 4% paraformaldehyde (PFA)/PBS treated for 15 min, and permeabilized with 0.1% Triton X-100/PBS for 10 min. F-actin/Phalloidin (1:1,000; Cat. #: A22287, ThermoFisher Scientific) was used to incubate the coverslips for one hour. DAPI (Cat. #: D3571, Invitrogen) was used to visualize nuclei. Images were taken using Olympus BX43F microscope. Gelatin degradation was quantified using ImageJ software. To measure the percentage of degraded area in each field, identical signal threshold for the Oregon Green -gelatin fluorescence is set for all images and the degraded area with Oregon Green signal below the set threshold was measured by ImageJ. The resulting percentage of degradation area was further normalized to total number of cells (counted by DAPI staining for nuclei) in each field. The final gel degradation index is the average percentage degradation per cell obtained from fields.

### Protein stability assay

To examine cortactin protein turnover, cycloheximide (0.1 mg/ml) was added to cell culture medium and cells were harvested at the indicated time points. Cells were then lysed in RIPA lysis buffer and cell lysates were subjected to Western blot with anti‐cortactin, anti‐USP43, or anti-Ca_v_2.2 and anti‐β‐actin as indicated.

### In vivo deubiquitination assay

MDA-MB-231 cells were treated with MG132 for 6 h before harvesting. The cells were lysed in 800 μl IP-lysis buffer containing protease inhibitors, 20 mM NEM, and 1 mM iodoacetamide, centrifuged to remove cell debris. The cell extracts were subjected to immunoprecipitation with Flag-beads at 4 °C. After washing with IP lysis buffer (containing 20 mM NEM and 1 mM iodoacetamide) four times, the immunocomplexes were separated by SDS–PAGE and blotted with anti‐Ub antibody.

### Co‐immunoprecipitation (co‐IP) assay

Cells were harvested and washed by PBS before lysed with IP lysis buffer (Meilunbio, China) with 1 mM PMSF. Whole cell lysates obtained by centrifugation were incubated with 2 μg of anti-Flag-beads (sigma) at 4 °C. After washing with IP lysis buffer three times, the immunocomplexes were separated by SDS–PAGE. Immunoblotting was performed following standard procedures.

### Measurement of intracellular calcium

Cells were loaded with calcium-sensitive fluorescent dye 1 μM Fura-2AM (Beyotime) for 40 min at room temperature in the dark and then washed three times in standard external solution of 145 mM NaCl, 2.8 mM KCl, 2 mM CaCl_2_, 2 mM MgCl_2_, 10 mM D- glucose, 10 mM HEPES, pH 7.4, with NaOH. Fluorescence was measured with fluorescence spectrophotometer by varioskan LUX (Thermo Fisher Scientific), where excitation was performed at 340 nm and 380 nm. The fluorescence intensity (F340/F380) indicated an intracellular calcium concentration.

### Dual luciferase reporter assay

USP43 promoter was cloned into pGL3 basic plasmid to construct pGL3-USP43 promoter-Luc plasmid. MDA-MB-231 cells were cultured in 12-well plates. MDA-MB-231 cells expressing control vector or NFAT2 were co-transfected 500 ng USP43 promoter-Luc plasmid and 20 ng Renilla using lipofectamine 2000 (Invitrogen). Firefly and Renilla luciferase activities were measured using the dual-luciferase reporter assay system 48 h after transfection (Cat:#E1910, Promega).

### Chromatin immunoprecipitation (ChIP)

ChIP were performed according to manufacture’s protocol (Cat:#53040,Active motif). Briefly, two 10 cm dishes MDA-MB-231 cells were cross-linked and lysed. Cells were sonicated using the Bioruptor Pico (Diagenode) for 30 s on/30 s off for 15 cycles. Chromatin was immunoprecipitated with the specific antibody at 4 °C overnight, followed by incubation with protein A/G beads for 3 h at 4 °C. The immune complex was then eluted from beads and was reversed cross-linked by incubating in elution buffer. The DNA fragments were purified and used for qPCR using SYBR Green Master Mix.

### Immunofluorescence staining

Cells were plated on the glass slides in 12-wells. Then cells were fixed with 4% paraformaldehyde for 15 min at room temperature. After being washed twice with PBS, the cells were permeabilized with 0.1% Triton-X100 for 30 min and blocked with 5% BSA for 1 h at room temperature. Cells were incubated with a primary antibody against NFAT2 at the ratio of 1:300 at 4 °C overnight, and subsequently incubated with a secondary antibody at room temperature for 1 h, followed by mounting with Vectashield Mounting Media with DAPI (H-1200, Vector Laboratories). Images were taken using Olympus BX43F microscope and analyzed with ImageJ.

## Results

### Identification of Ca_v_2.2 in breast cancer progression

**Fig. 1:**
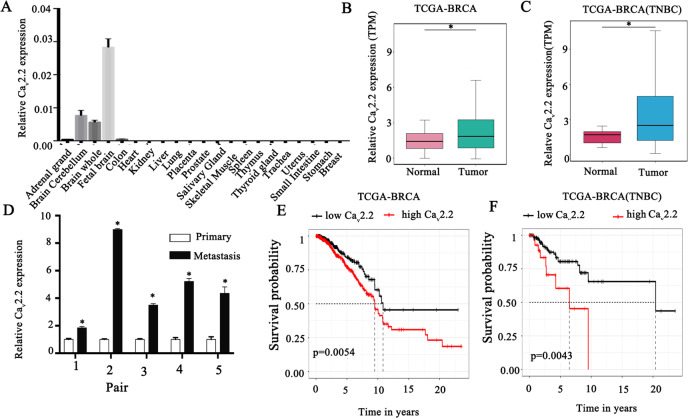
Cav2.2 is expressed in human breast cancer tissues but not in normal human breast tissues. **A** Expression of Ca_v_2.2 transcript in normal human tissues. Error bars represent mean ± sd **B** Expression of Cav2.2 transcript in breast cancer tissues and normal tissues. *p < 0.05. **C** Expression of Cav2.2 transcript in triple negative breast cancer (TNBC) tissues and adjacent normal tissues. *p < 0.05. **D** Expression of Cav2.2 transcript in paired metastatic and primary human breast cancer tissues. *p < 0.02. **E** Association of higher Cav2.2 expression with poor survival in breast cancer (Cox regression *P* = 0.0054, hazard ratio = 1.58). **F** Association of higher Cav2.2 expression with poor survival in triple negative breast cancer (Cox regression *P* = 0.0043, hazard ratio = 2.95).

To identify critical genes that drive breast cancer progression, we analyzed The Cancer Genome Atlas (TCGA) RNA-seq data of breast cancer and normal breast tissues and the relevant survival data of breast cancer patients [38]. From the analysis, 41 genes were identified that met the four inclusion criteria as described previously [38]. In this study, we focused on the calcium channel Ca_v_2.2 because (1) very little is known about its functions in cancer development; (2) it is expressed in breast cancer but not in normal tissues other than brain; (3) it is a cell surface protein which can be targeted by small molecule compounds or antibodies; (4) FDA-approved therapeutics targeting Ca_v_2.2 is already used in clinics for chronic pain. We first determined the Ca_v_2.2 expression in a panel of normal human tissues and found that it is exclusively expressed in adult and fetal brain tissues (Fig. 1A). Interestingly, Ca_v_2.2 expression was upregulated in breast cancer tissues than adjacent normal breast tissues (Fig. 1B). In particular, Ca_v_2.2 expression was significantly upregulated in triple negative breast cancer (TNBC) (Fig. 1C), which has worse prognosis than other subtypes in breast cancer. Consistent with the Ca_v_2.2 expression in breast cancer tissues, Ca_v_2.2 was expressed at various levels in breast cancer cell lines but not in human normal breast epithelial cell line HMEL (Supplementary Fig. 1). In paired human breast cancer samples, Ca_v_2.2 expression was significantly higher in the metastatic samples than in the matched primary breast cancer samples (Fig. 1D). We further investigated the correlation of Ca_v_2.2 expression with survival. Patients with higher Ca_v_2.2 expression had worse overall survival than those with lower Ca_v_2.2 expression in breast cancer (Fig. 1E) and the TNBC subtype (Fig. 1F). Taken together, these data indicated that Ca_v_2.2 expression is up-regulated in breast cancer and a potential prognosis marker for survival.

### Ca_v_2.2 is required for breast cancer invasion and metastasis

**Fig. 2:**
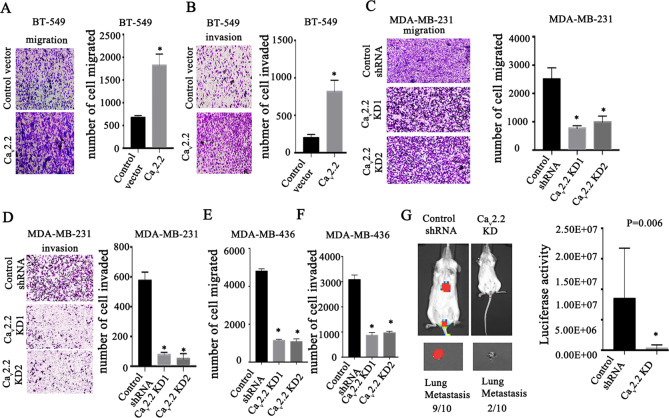
Cav2.2 is required for migration, invasion and metastasis in breast cancer. Human breast cancer BT-549 cells transfected with Cav2.2 cDNA or a control vector were subjected to migration **A** and invasion **B** assays. Representative images of migrated or invaded cells were shown. The numbers of migrated and invaded cells were quantified. P value was determined using Student’s t-test (*p < 0.001). Error bars represent mean ± s.d. Human breast cancer MDA-MB-231 cells stably expressing Cav2.2 shRNA or control shRNA were subjected to migration **C** and invasion **D** assays. Representative images of migrated or invaded cells were shown. The numbers of migrated and invaded cells were quantified. *P* value was determined using Student’s t-test (*p < 0.001). Error bars represent mean ± sd. Human breast cancer MDA-MB-436 cells stably expressing Cav2.2 shRNA or control shRNA were subjected to migration **E** and invasion **F** assays. The numbers of migrated and invaded cells were quantified. *P* value was determined using Student’s t-test (*p < 0.001). Error bars represent mean ± sd. **G** Luciferase-tagged human breast cancer MDA-MB-436 cells stably expressing Cav2.2 shRNA or control shRNA were transplanted in mice. Metastasis were imaged using Xenogen bioluminescence system. *P* value was determined using Fisher’s exact test (*p* = 0.006).

As Ca_v_2.2 appeared to have higher expression in metastatic tissues than in primary tumors and metastasis is a major factor in patient survival, we assessed the role of Ca_v_2.2 in breast cancer invasion and metastasis. We introduced Ca_v_2.2 into human breast cancer cell BT549 which expresses endogenous Ca_v_2.2 at a very low level. The expression of Ca_v_2.2 in BT549 was confirmed by real-time PCR (Supplementary Fig. 2). The cells were subjected to transwell assays. Ca_v_2.2 significantly increased cell migration and invasion (Fig. 2A, B) without affecting cell proliferation (Supplementary Fig. 3). To investigate whether Ca_v_2.2 is required for migration and invasion, we introduced short hairpin RNA constructs into human breast cancer cell lines MDA-MB-231 and MDA-MB-436. Knock-down of Ca_v_2.2 expression was confirmed by real-time PCR (Supplementary Figs. 4 and 5). Ca_v_2.2 knock-down significantly reduced cell migration and invasion in MDA-MB-231 and MDA-MB-436 (Fig. 2C–F) without affecting cell growth (Supplementary Fig. 6, Supplementary Fig. 7). To investigate the function of Ca_v_2.2 in metastasis, we knocked down Ca_v_2.2 in luciferase-tagged MDA-MB-436 cells and transplanted these cells into mice. Nine out of ten mice transplanted with cells expressing a control shRNA construct developed metastasis (Fig. 2G), only two out of ten mice transplanted with cells expressing Ca_v_2.2 shRNA developed metastasis (Fig. 2G). The luciferase signal of metastasis was also much higher in these nine mice (Fig. 2G). These results indicated that Ca_v_2.2 was required for cell migration, invasion, and metastasis.

### Ca_v_2.2 promotes invadopodia formation and extracellular matrix degradation

**Fig. 3:**
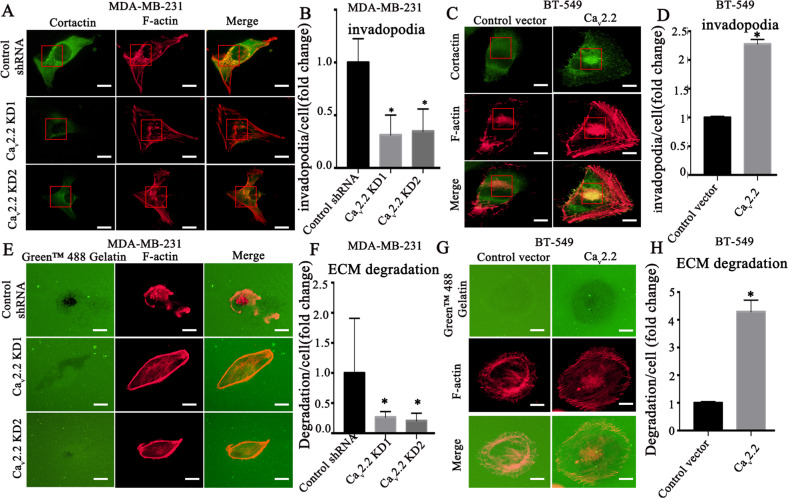
Cav2.2 promotes invadopodia formation and ECM degradation. **A** Human breast cancer MDA-MB-231 cells stably expressing Ca_v_2.2 shRNA or control shRNA were subjected to invadopodia formation assay. Invadopodia were visualized by co-localization of cortactin (green) and F-actin puncta (red). Representative images were shown. Bars: 5 μm. **B** Quantification of invadopodia in MDA-MB-231 cells. *P* value was determined using Student’s t-test (*p < 0.01). Error bars represent mean ± s.d. **C** Human breast cancer BT-549 cells transfected with Ca_v_2.2 cDNA or a control vector were subjected to invadopodia formation assay. Invadopodia were visualized by co-localization of cortactin (green) and F-actin puncta (red). Representative images were shown. Bars: 5 μm. **D** Quantification of invadopodia in BT-549 cells. *P* value was determined using Student’s t-test (*p < 0.01). Error bars represent mean ± sd. **E** MDA-MB-231 cells stably expressing Ca_v_2.2 shRNAs or control shRNA were subjected to ECM degradation assay. Focal matrix degradation was defined by co-localization of F-actin and degradation regions. Representative images were shown. Bars: 5 μm. **F** Quantification of ECM degradation in MDA-MB-231 cells. P value was determined using Student’s t-test (*p < 0.01). Error bars represent mean ± ^s.d.^ **G** BT-549 cells transfected with Ca_v_2.2 cDNA or a control vector were subjected to ECM degradation assay. Focal matrix degradation was defined by co-localization of F-actin and degradation regions. Representative images were shown. Bars: 5 μm. **H** Quantification of ECM degradation in BT-549 cells. *P* value was determined using Student’s t-test (*p < 0.01). Error bars represent mean ± sd.

The invasiveness of cancer cells depends on their ability to degrade extracellular matrix (ECM) and invade into neighboring tissues as well as lymph node and bloodstream [12, 39]. Their invasiveness is decided by special cell membrane structure invadopodia which are dynamic actin‐enriched cell protrusions with proteolytic activity. Co-localization of F-actin with the actin-bundling protein cortactin can be used to identify invadopodia [40]. To dissect the cellular functions of Ca_v_2.2 in invadopodia formation, Ca_v_2.2 knock-down or over-expression cells were plated on gelatin matrix and labeled for F-actin and cortactin. We found that knock-down of Ca_v_2.2 potently suppressed the ability of MDA-MB-231 cells to form invadopodia (Fig. 3A, B). Conversely, Ca_v_2.2 overexpression significantly increased invadopodia formation in BT-549 cells (Fig. 3C, D). Interestingly, immunofluorescence also showed that Cav2.2 knock-down significantly decreased fluorescence intensity of cortactin (Supplementary Fig. 8A), whereas Ca_v_2.2 overexpression significantly increased cortactin fluorescence intensity (Supplementary Fig. 8B). These results suggested that Ca_v_2.2 modulates cortactin protein expression. Since functional invadopodia are able to degrade localized ECM, we determined whether Ca_v_2.2 was required to accelerate focal matrix degradation by gelatin degradation assay. Indeed, Ca_v_2.2 knock-down cells showed a significantly reduced capability to degrade focal ECM (Fig. 3E, F). Conversely, expression of Ca_v_2.2 led to dramatically increased degradation of focal ECM (Fig. 3G, H). Taken together, these data demonstrated that Ca_v_2.2 is sufficient and required for invadopodia formation and ECM degradation.

### Ca_v_2.2 stabilizes cortactin in a proteosome-dependent manner

**Fig. 4:**
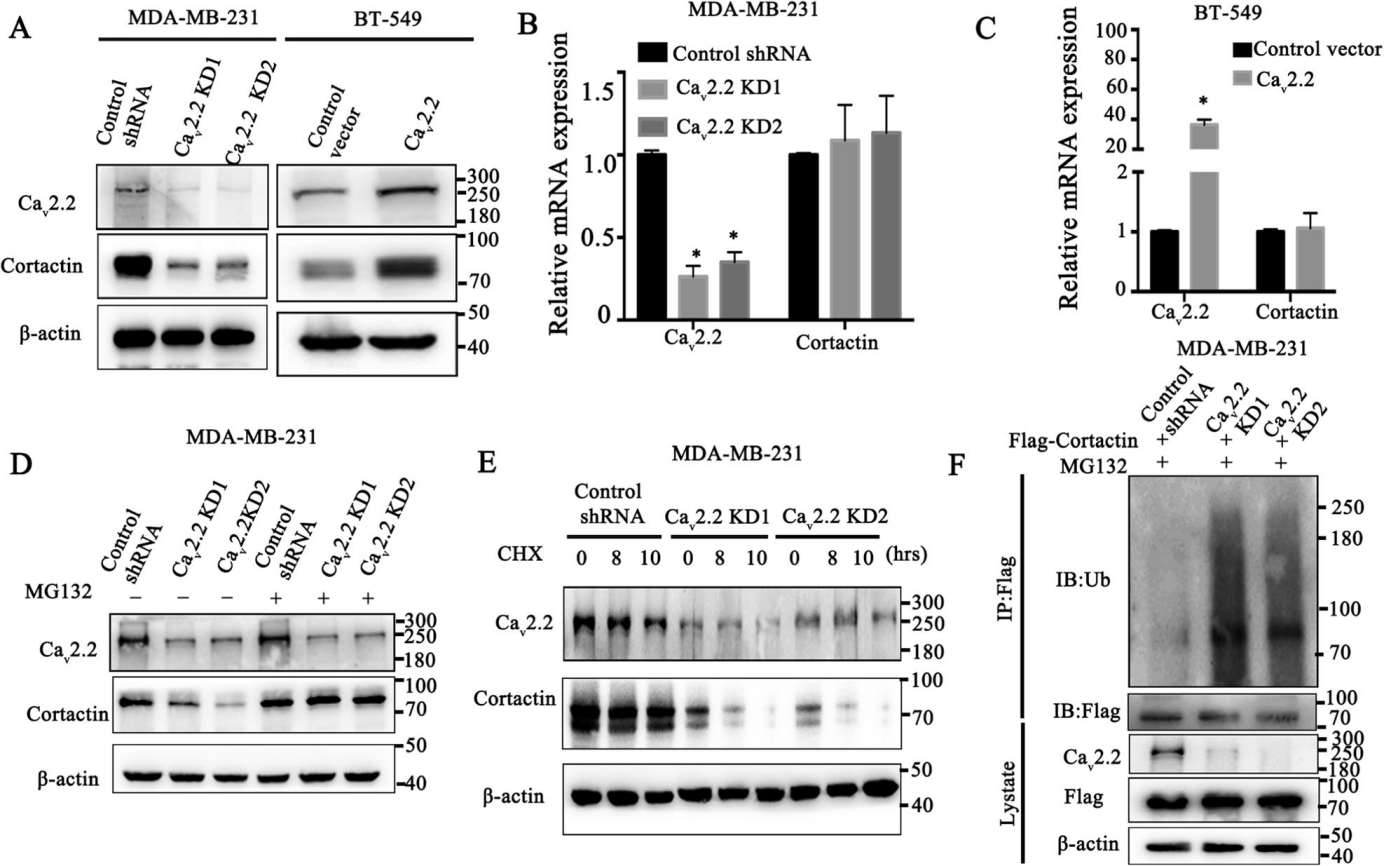
Cav2.2 stabilizes cortactin in a proteosome-dependent manner. **A** Ca_v_2.2 and cortactin were determined by immunoblotting in MDA-MB-231 cells stably expressing Ca_v_2.2 shRNA or control shRNA and BT-549 cells transfected with Ca_v_2.2 cDNA or a control vector. **B** Ca_v_2.2 and cortactin transcripts were determined by qRT-PCR in MDA-MB-231 cells stably expressing Ca_v_2.2 shRNA or control shRNA. P value was determined using Student’s t-test (*p < 0.001). Error bars represent mean ± s.d. **C** Ca_v_2.2 and cortactin transcripts were determined by qRT-PCR in BT-549 cells transfected with Ca_v_2.2 cDNA or control vector. P value was determined using Student’s t-test (*p < 0.001). Error bars represent mean ± s.d. **D** Ca_v_2.2 and cortactin were determined by immunoblotting in MDA-MB-231 cells stably expressing Ca_v_2.2 shRNA or control shRNA and treated with proteasome inhibitor MG132 or a control vehicle. **E** Ca_v_2.2 and cortactin were determined by immunoblotting in MDA-MB-231 cells stably expressing Ca_v_2.2 shRNA or control shRNA and treated with protein synthesis inhibitor cycloheximide (CHX) or a control vehicle. **F** MDA-MB-231 cells stably expressing Flag-tagged cortactin and Ca_v_2.2 shRNA or control shRNA were treated with proteosome inhibitor MG132. Ubiquitination of cortactin were determined by immunoblotting of ubiquitin following Flag-tag immunoprecipitation.

To gain insight into the underlying mechanism of Ca_v_2.2 in the regulation of invadopodia formation, we examined whether Ca_v_2.2 regulated the cortactin expression given that cortactin plays a key role in the formation of invadopodia and invadopodia associated ECM degradation [41–43] and our immunofluorescence analysis showed Ca_v_2.2 regulated cortactin expression. Immunoblotting analysis showed that the cortactin protein expression was greatly decreased after Ca_v_2.2 knock-down in MDA-MB-231 cells (Fig. 4A), whereas the cortactin transcript was not changed in Ca_v_2.2 knock-down cells (Fig. 4B). Conversely, expression of Ca_v_2.2 in BT-549 cells resulted in a significant increase of cortactin protein expression (Fig. 4A), but not cortactin transcript (Fig. 4C). These results suggested a mechanism involving post-transcriptional regulation of cortactin. Ubiquitination and deubiquitination are among the most widely-used protein modifications in regulating cellular signaling and homeostasis [44]. Because ubiquitination generally modulates target expression by inducing proteasome‐dependent degradation [45], we added the proteasome inhibitor MG132 into MDA-MB-231 cells expressing Ca_v_2.2 shRNA or control shRNA. We found that the decrease in cortactin expression by Ca_v_2.2 knock-down could be reversed by the addition of MG132, suggesting that Ca_v_2.2 regulates cortactin in a proteasome‐dependent manner (Fig. 4D). To further assess the effects of Ca_v_2.2 on cortactin stability, we added cycloheximide (CHX) which suppresses protein synthesis and monitor the degradation of cortactin protein. As shown in Fig. 4E, cortactin stability was dramatically decreased in Ca_v_2.2 knock-down cells. These results demonstrated that Ca_v_2.2 stabilizes cortactin in breast cancer cells. We next examined whether Ca_v_2.2 regulated cortactin ubiquitination or deubiquitination in breast cancer cells. As shown in Fig. 4F, knock-down of Ca_v_2.2 in MDA-MB-231 cells strongly increased the ubiquitination of cortactin. These data demonstrated that Ca_v_2.2 modulated the protein stability of cortactin through ubiquitination or deubiquitination process in breast cancer cells.

### Identification of USP43 as a deubiquitinating enzyme for cortactin stability

**Fig. 5:**
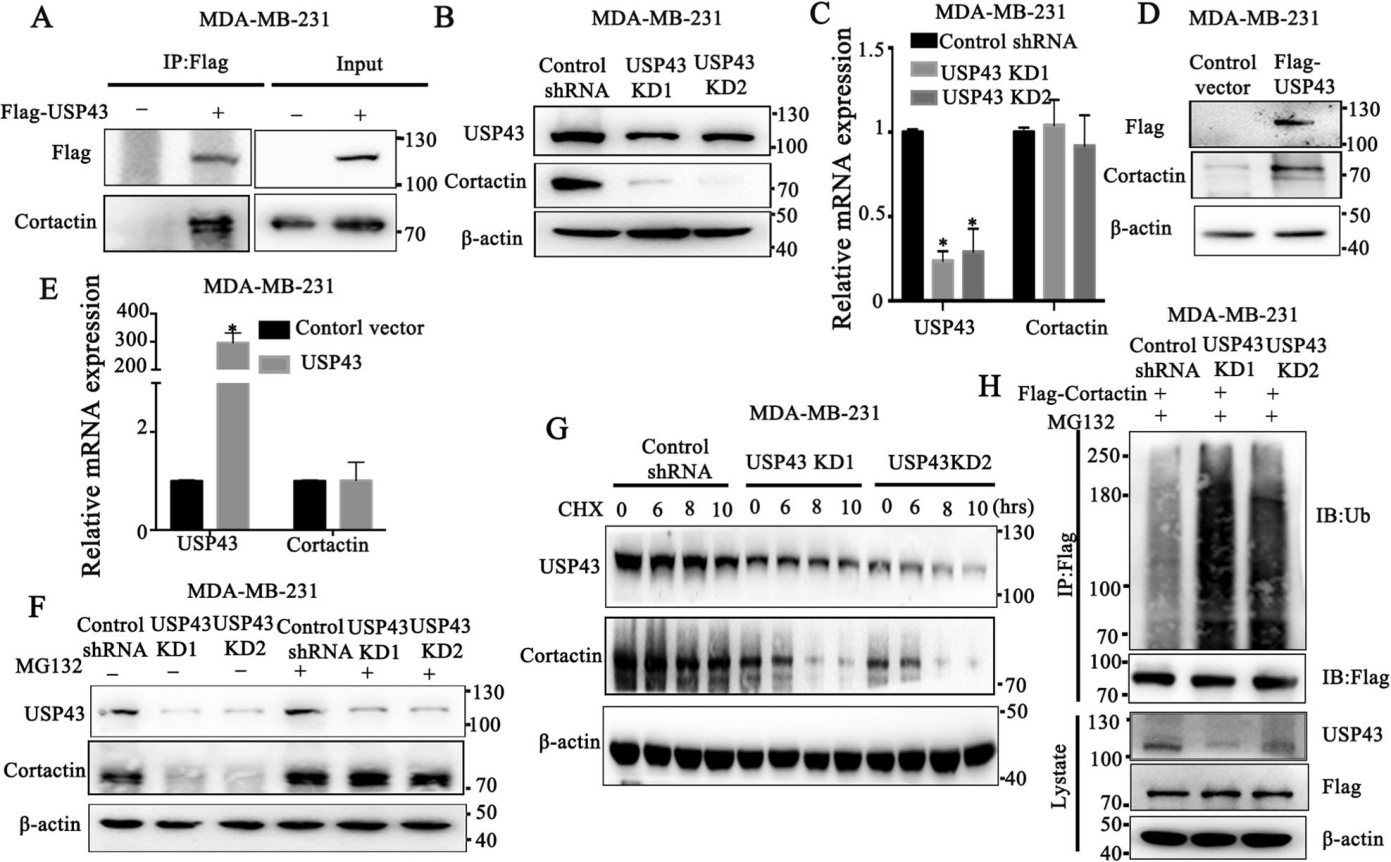
USP43 interacts with and deubiquitinates cortactin. **A** Cortactin were determined by immunoblotting in MDA-MB-231 cells expressing Flag-tagged USP43 or control vector following Flag-tag immunoprecipitation. **B** Cortactin were determined by immunoblotting in MDA-MB-231 cells expressing USP43 shRNA or control shRNA. **C** Cortactin and USP43 transcripts were determined by qRT-PCR in MDA-MB-231 cells expressing USP43 shRNA or control shRNA. P value was determined using Student’s t-test (*p < 0.001). Error bars represent mean ± s.d. **D** Cortactin were determined by immunoblotting in MDA-MB-231 cells expressing flag-tagged USP43 cDNA or a control vector. **E** Cortactin and USP43 transcripts were determined by qRT-PCR in MDA-MB-231 cells expressing USP43 cDNA or a control vector. **F** Cortactin and USP43 were determined by immunoblotting in MDA-MB-231 cells stably expressing USP43 shRNA or control shRNA and treated with proteosome inhibitor MG132 or a control vehicle. **G** Cortactin and USP43 were determined by immunoblotting in MDA-MB-231 cells stably expressing USP43 shRNA or control shRNA and treated with protein synthesis inhibitor cycloheximide (CHX) or a control vehicle. **H** MDA-MB-231 cells stably expressing Flag-tagged cortactin and USP43 shRNA or control shRNA were treated with proteosome inhibitor MG132. Ubiquitination of cortactin determined by immunoblotting of ubiquitin following Flag-tag immunoprecipitation.

As Ca_v_2.2 is a calcium channel protein located on the cell surface, it is unlikely that it directly deubiquitinates cortactin. To gain insight how Ca_v_2.2 decreased cortactin ubiquitination, we analyzed the correlation between the expression of cortactin and ubiquitin ligases or deubiquinating enzymes by using the proteomics data from Cancer Cell Line Encyclopedia (CCLE) database. Candidates from the analysis should satisfy the following criteria: (1) p value and q value below 0.01; (2) the Pearson correlation coefficient above zero if the candidate is a deubiquitinase. Conversely, Pearson correlation coefficient below zero if the candidate is a E3 ligase. Only six deubiquitinating enzymes (DUBs), USP43, OTUD1, OTUD4, OTUD7A, USP40, USP54 met our criteria. We then transfected the cDNAs of these six DUBs into MDA-MB-231 cells, and immunoblotting showed that only USP43 increased cortactin expression (Supplementary Fig. 9A). The expressions of these six DUBs were confirmed by real-time PCR (Supplementary Fig. 9B–G). We first determined whether USP43 interacted with cortactin. Immunoprecipitation showed that USP43 interacted with cortactin in MDA-MB-231 cells (Fig. 5A). Knock-down of USP43 in MDA-MB-231 cells decreased the expression of cortactin at the protein level (Fig. 5B) but did not affect its transcript (Fig. 5C). Conversely, over-expression of USP43 increased cortactin protein expression without affecting its expression at the transcriptional level (Fig. 5D, E). The addition of MG132 successfully rescued the decrease of cortactin expression caused by USP43 knock-down (Fig. 5F), while the addition of CHX resulted in more unstable cortactin protein in USP43 knock-down cells (Fig. 5G). These results demonstrated that USP43 stabilized cortactin protein in MDA-MB-231 cells. We next investigated whether USP43 was able to modulate cortactin ubiquitination in MDA-MB-231 cells. Knock-down of USP43 increased the addition of ubiquitin on cortactin (Fig. 5H). Together, these results suggested that USP43 directly regulates cortactin stability through deubiquitination.

### USP43 mediates the functions of Ca_v_2.2 in cortactin stabilization, invadopodia formation, and metastasis in breast cancer

**Fig. 6:**
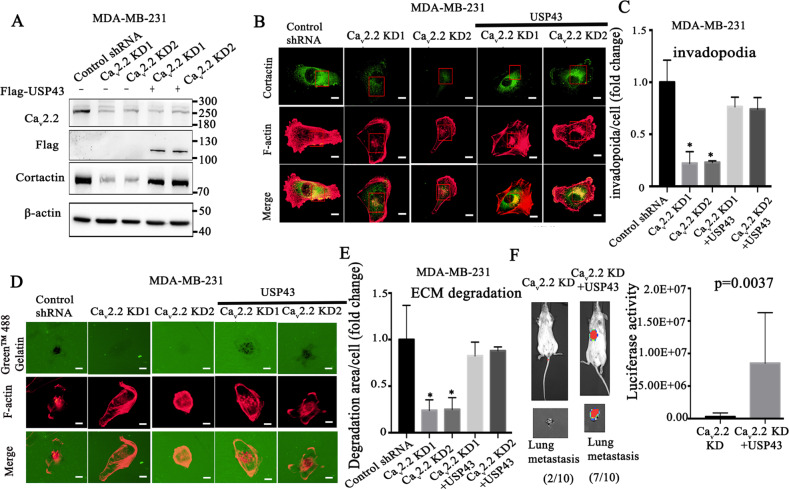
USP43 mediates the function of Cav2.2 in cortactin stabilization, invadopodia formation, ECM degradation and metastasis in breast cancer. **A** Ca_v_2.2 and cortactin were determined by immunoblotting in MDA-MB-231 cells expressing Flag-tagged USP43 and Ca_v_2.2 shRNA or control shRNA. **B** MDA-MB-231 expressing USP43 cDNA and Ca_v_2.2 shRNA or control shRNA were subjected to invadopodia formation assay. Invadopodia were visualized by co-localization of cortactin (green) and F-actin puncta (red). Representative images were shown. Bars: 5 μm. **C** Quantification of invadopodia in MDA-MB-231 cells. *P* value was determined using Student’s t-test (*p < 0.01). Error bars represent mean ± s.d. **D** MDA-MB-231 cells expressing USP43 cDNA and Ca_v_2.2 shRNAs or control shRNA were subjected to ECM degradation assay. Focal matrix degradation was defined by co-localization of F-actin and degradation regions. Representative images were shown. Bars: 5 μm. **E** Quantification of ECM degradation in MDA-MB-231 cells. *P* value was determined using Student’s t-test (*p < 0.01). Error bars represent mean ± s.d. **F** Luciferase-tagged human breast cancer MDA-MB-436 cells stably expressing Cav2.2 shRNA or Cav2.2 shRNA and USP43 cDNA were transplanted in mice. Luciferase signal of metastasis was quantified using Xenogen bioluminescence system. *P* value was determined using Fisher’s exact test (*p* = 0.0037).

To determine whether USP43 mediated the functions of Ca_v_2.2, we introduced Ca_v_2.2 shRNA and USP43 cDNA constructs into breast cancer cells. Immunoblotting showed that cortactin expression was stabilized in Ca_v_2.2 knock-down cells expressing USP43 (Fig. 6A), indicating USP43 mediated the function of Ca_v_2.2 in cortactin stabilization. We further investigated whether USP43 mediated Ca_v_2.2 function in invadopodia formation, we expressed USP43 cDNA in Ca_v_2.2 knock-down cells and found that USP43 expression rescued the decrease of invadopodia formation caused by Ca_v_2.2 knock-down (Fig. 6B, C). Similarly, focal matrix degradation was also rescued in USP43 cDNA expression cells (Fig. 6D, E). To further investigate if USP43 mediated the function of Ca_v_2.2 in metastasis in vivo, we transplanted luciferase-tagged MDA-MB-436 cells stably expressing Ca_v_2.2 shRNA and USP43 cDNA constructs into mice. Seven out of ten mice transplanted with cells expressing Ca_v_2.2 shRNA and USP43 developed metastases, whereas two out of ten mice expressing Ca_v_2.2 shRNA developed metastasis (Fig. 6F). The luciferase signal of metastasis was also significantly higher in mice injected with cells expressing Ca_v_2.2 shRNA and USP43 (Fig. 6F). These results indicated that USP43 mediated the functions of Ca_v_2.2 in cortactin expression, invadopodia formation, ECM degradation, and metastasis.

### USP43 is directly regulated by NFAT2

**Fig. 7:**
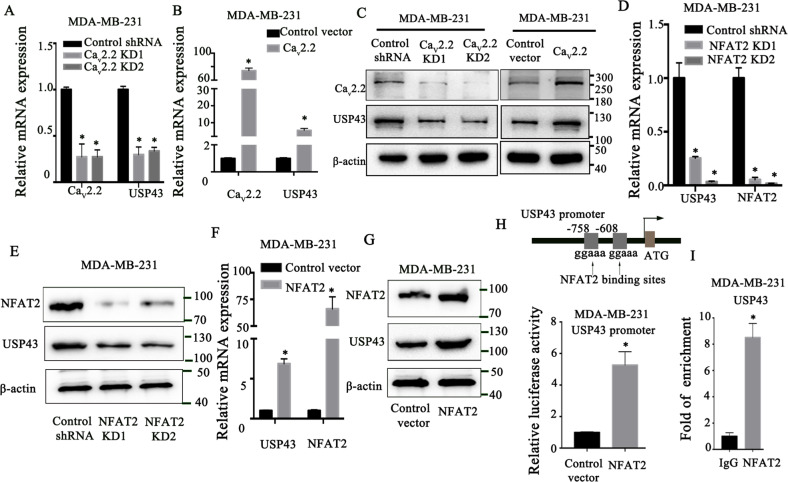
USP43 is directly regulated by NFAT2. **A** Ca_v_2.2 and USP43 expression were determined by qRT-PCR in MDA-MB-231 cells stably expressing Ca_v_2.2 shRNA or a control shRNA. P value (*p < 0.001) was determined using Student’s t-test. Error bars represent mean ± s.d. **B** Ca_v_2.2 and USP43 expression were determined by qRT-PCR in MDA-MB-231 cells expressing Ca_v_2.2 cDNA or a control vector. **C** USP43 and Ca_v_2.2 expression were determined by immunoblotting in MDA-MB-231 cells stably expressing Ca_v_2.2 shRNA or a control shRNA, Ca_v_2.2 cDNA or a control vector. **D** USP43 and NFAT2 expression were determined by qRT-PCR in MDA-MB-231 cells expressing NFAT2 shRNA or a control shRNA. P value (*p < 0.001) was determined using Student’s t-test. Error bars represent mean ± s.d. **E** NFAT2 and USP43 expression were determined by immunoblotting in MDA-MB-231 cells stably expressing NFAT2 shRNA or a control shRNA. **F** USP43 and NFAT2 expression were determined by qRT-PCR in MDA-MB-231 cells expressing NFAT2 cDNA or a control vector. P value (*p < 0.001) was determined using Student’s t-test. Error bars represent mean ± s.d. **G** NFAT2 and USP43 expression were determined by immunoblotting in MDA-MB-231 cells stably expressing NFAT2 cDNA or a control vector. **H** NFAT2 binding sites on the USP43 promoter were showned (top). The luciferase activity of USP43-promoter was determined by dual lucifierase reporter assay in MDA-MB-231 cells expressing pGL3-USP43 promoter-Luc plasimid and NFAT2 cDNA or a control vector (bottom). Luciferase activity values were normalized to renilla luciferase activity values and expressed as fold change over a control vector. P value (*p < 0.001) was determined using Student’s t-test. Error bars represent mean ± s.d. **I** Chromatin immunoprecipitation (ChIP) was performed using an anti-NFAT2 antibody or a control IgG. The USP43 promoter region where NFAT2 binds showed significant enrichment after immunoprecipitation by an anti-NFAT2 antibody. *P* value (*p < 0.001) was determined using Student’s t-test. Error bars represent mean ± s.d.

We then investigated the regulation of USP43 by Ca_v_2.2 and found that knock-down of Ca_v_2.2 decreased USP43 expression whereas over-expression of Ca_v_2.2 increased USP43 expression transcriptionally and at the protein level (Fig. 7A–C). These results showed that Ca_v_2.2 is sufficient and required for USP43 expression. We further explored the molecular mechanism of this regulation. Because Ca_v_2.2 is a calcium channel, we first tested whether [Ca^2+^]i (intracellular calcium level) was changed upon the decrease or increase of Ca_v_2.2 expression. [Ca^2+^]i was significantly decreased in Ca_v_2.2 knock-down cells (Supplementary Fig. 10). Conversely, [Ca^2+^]i level was significantly elevated in Ca_v_2.2 overexpression cells (Supplementary Fig. 10). These results confirmed that Ca_v_2.2 regulates calcium influx. Calcium influx activates heterodimeric phosphatase calcineurin, which dephosphorylates NFAT family proteins (NFAT1, NFAT2, NFAT3, NFAT4) andresults in their nuclear translocation [46]. Nuclear NFAT family proteins activate target gene expression, thus directly linking calcium signaling to gene expression [46]. We then investigated whether NFAT proteins were able to modulate USP43 mRNA expression. We found that depletion of NFAT2 and NFAT3 could significantly reduced USP43 mRNA expression (Fig. 7D, Supplementary Fig. 11). However, the knock-down of NFAT1 and NFAT4 did not decrease USP43 mRNA expression (Supplementary Fig. 12). We further determined the USP43 protein expression in NFAT2 or NFAT3 knock-down cells. NFAT2 or NFAT3 knock-down resulted in the decrease of USP43 protein expression (Fig. 7E, Supplementary Fig. 13). The ChIP analysis showed that NFAT3 did not bind to the NFAT consensus sequence in the USP43 promoter region we studied, but binded to the BACE1 promoter region which is a well-established direct target gene of NFAT3 and served as a control [47] (Supplementary Fig. 14A, Supplementary Fig. 14B). It is possible that NFAT3 binds to the other regions of USP43 promoter or indirectly regulates USP43 expression. Since we intend to find a transcription factor that directly regulates USP43 expression, we chose to focus on NFAT2 for further study. We transduced NFAT2 cDNA into MDA-MB-231 cells. Expression of NFAT2 increased USP43 mRNA and protein expression (Fig. 7F, G). These results suggested that USP43 is regulated by NFAT2. To determine whether USP43 is a direct target of NFAT2, we found two consensus binding sites (ggaaa) of NFAT2 in the USP43 promoter region (Fig. 7H) [48]. To verify whether USP43 is directly regulated by NFAT2, we cloned USP43 promoter (−213~−1847bp) into pGL3-basic vector and performed dual luciferase reporter assay. NFAT2 expression significantly increased the luciferase signal driven by the USP43 promoter (Fig. 7H). We used Chromatin Immunoprecipitation (ChIP) analysis to further determined the direct interactions of NFAT2 with USP43 promoter in MDA-MB-231 cells, ChIP analysis indicated the significant enrichment of USP43 promoter as well as BMI1 promoter which is a well-established direct target gene of NFAT2 [49] and served as a control in NFAT2 immunoprecipitation (Fig. 7I, Supplementary Fig. 15). These data suggested that NFAT2 directly regulates USP43 transcription.

### Ca_v_2.2 regulates USP43 expression through NFAT2 dephosphorylation

**Fig. 8:**
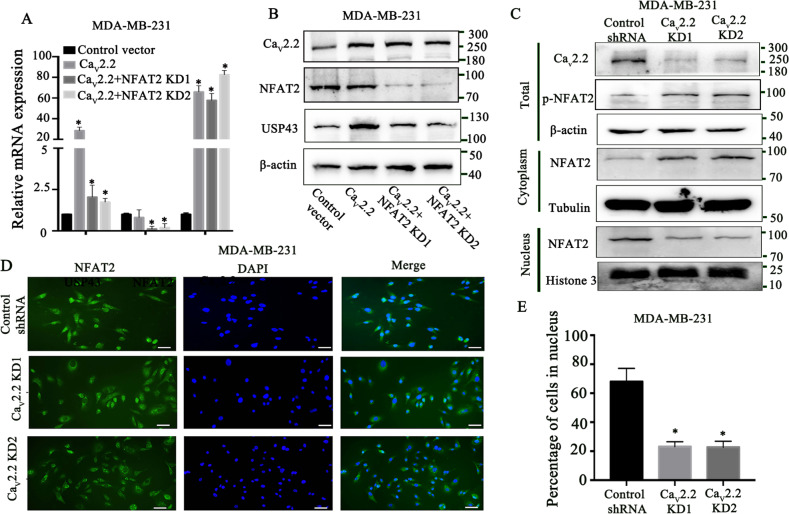
Cav2.2 regulates USP43 expression through NFAT2 dephosphorylation. **A** USP43, NFAT2, and Ca_v_2.2 expression were determined by qRT-PCR in MDA-MB-231 cells expressing Ca_v_2.2 cDNA and NFAT2 shRNA or a control shRNA. P value was determined using Student’s t-test (*p < 0.001). Error bars represent mean ± s.d. **B** Ca_v_2.2, NFAT2, and USP43 expression were determined by immunoblotting in MDA-MB-231 cells expressing Ca_v_2.2 cDNA and NFAT2 shRNA or a control shRNA. **C** Ca_v_2.2 and phosphorylated NFAT2 expression were determined by immunoblotting in MDA-MB-231 cells expressing Ca_v_2.2 shRNA or a control shRNA. NFAT2 expression in cyltoplasm and nucleus were determined by immunoblotting in MDA-MB-231 cells expressing Ca_v_2.2 shRNA or a control shRNA. **D** The subcellular localization of NFAT2 was determined by immunofluorescence in MDA-MB-231 cells expressing Ca_v_2.2 shRNA or a control shRNA. Representative images were shown. Bars: 20 μm. **E** Quantification of NFAT2 nuclear abundance in MDA-MB-231 cells expressing Ca_v_2.2 shRNA or a control shRNA. *P* value was determined using Student’s t-test (*p < 0.01). Error bars represent mean ± s.d.

We further investigated whether NFAT2 mediates the function of Ca_v_2.2 in USP43 regulation. We knocked down NFAT2 expression in Ca_v_2.2 over-expression cells and determined USP43 expressions. NFAT2 knock-down abolished the up-regulation of USP43 levels by Ca_v_2.2 (Fig. 8A, B). These data indicated that NFAT2 mediates the regulation of USP43 by Ca_v_2.2. We next determined the molecular mechanism of NFAT regulation by Ca_v_2.2. Ca_v_2.2 knockdown increased the phosphorylation of NFAT2 and its cytoplasmic localization, while decreased NFAT2 nuclear localization. (Fig. 8C–E). Together, these experiments suggested that Ca_v_2.2 is required for the direct regulation of USP43 by NFAT2.

## Discussion

**Fig. 9:**
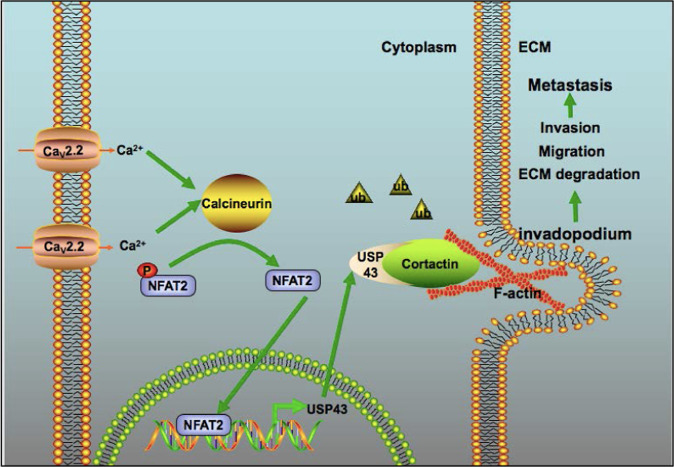
The working model of deubiquitinase USP43 mediating the functions of the calcium channel Cav2.2 in cortactin stabilization, invadopodia formation, and metastasis. Ca_v_2.2 up-regulates USP43 expression through NFAT2 dephosphorylation and nuclear localization. USP43 stablizes cortactin through deubiquitination and promotes invadopodia formation and metastasis.

The functions of calcium channels in tumor metastasis are poorly understood. Our study identified the role of calcium channel Ca_v_2.2 in breast cancer metastasis and its underlying mechanism (Fig. 9). Ca_v_2.2 was required for metastasis by promoting invadopodia formation. Surprisingly deubiquinating enzyme USP43 mediated the function of Ca_v_2.2 in invadopodia formation by stabilizing cortactin, a critical component of invadopodia. Strikingly, Ca_v_2.2 regulates USP43 mRNA and protein levels by activating calcineurin/NFAT2 signaling. To our knowledge, this is the first time calcium channel Ca_v_2.2 and deubiquitination process have been shown to play roles in invadopodia formation. Our study also revealed the functions of deubiquitinase USP43 in breast cancer cell migration, invasion, and metastasis. These studies uncovered a novel pathway that regulated invadopodia formation and metastasis in breast cancer.

Ca_v_2.2 is exclusively expressed in neuronal tissues but not in breast epithelial cells. The expression of a tissue-specific gene in the cancer of another tissue type presents a unique opportunity for therapy development. For an example, neuronal-specific glutamate receptor GRM1 is aberrantly expressed in melanoma and has been shown to play a critical role in melanoma development. GRM1 inhibitors as therapies for melanoma are currently in clinical trials. Ziconotide is an FDA-approved Ca_v_2.2 blocker for the treatment of severe and chronic pain. It is worthwhile to determine if ziconotide and other N-type calcium channel blockers can be used as therapies for the treatment of breast cancer metastasis. Development of novel Ca_v_2.2 blockers that are not able to cross blood–brain barrier may be even better for the treatment of breast cancer metastasis.

Invadopodia formation is required for breast cancer cells to degrade extracellular matrix and invade neighboring tissues [50]. However, invadopodia is difficult to target for therapy development because the main components of invadopodia F-actin and actin regulatory proteins are structurally undruggable. Our finding that deubiquiting enzyme USP43 regulates invadopodia formation may present a novel therapeutic target for the suppression of invadopodia and metastasis.

Our studies opened up a number of questions regarding the roles of Ca_v_2.2, NFAT2 and USP43 in invadopodia formation and metastasis. USP43 functions as a deubiquitinase. We have shown that cortactin is a substrate of USP43 in this study. Whether other factors in metastasis are also substrates of USP43 is unknown. The identification of substrates of USP43 that regulate invadopodia formation and metastatic process will improve our knowledge of the roles of ubiquiting process in metastasis. Cortactin regulation is also complex. In addition to the deubiquitination process we identified, it has been shown that phosphorylation can also regulate cortactin activity [36, 51]. The elucidation of the interplay between these regulatory processes in invasive cancer cells is critical to the understanding of tumor invasion and metastasis.

## Supplementary information

'previous manuscript for EA only

Supplementary Figure Legends

Checklist

Supplementary Figure

## Data Availability

The data supporting the findings of this study are available within the article and its supplementary information files.
