# Supplementary material for: Cav2.2-NFAT2-USP43 axis promotes invadopodia formation and breast cancer metastasis through cortactin stabilization
Source: Cell Death Dis. 2022 Sep 22;13(9):812. doi: 10.1038/s41419-022-05174-0 (PMC9500045; doi:10.1038/s41419-022-05174-0)
Supplement: Supplementary file 2 — Supplementary Figure Legends [file 41419_2022_5174_MOESM2_ESM.docx]

**Supplementary Figure Legends**

**Supplementary Figure 1**

Ca_v_2.2 expression were determined by immunoblotting in human breast cancer cell lines and normal breast epithelial cells.

**Supplementary Figure 2**

Ca_v_2.2 expression were determined by qRT-PCR in BT-549 cells expressing Ca_v_2.2 cDNA or a control vector. P value was determined using Student’s t-test (**p*<0.001). Error bars represent mean ± s.d.

**Supplementary Figure 3**

Human breast cancer BT-549 cells expressing Ca_v_2.2 cDNA or control vector were subjected to (A) CCK8 assay, (B) cell counting assay, (C) Ki-67 cell proliferation assay. The protein expression of Ki-67 was determined by immunofluorescence in BT549 cells expressing Ca_v_2.2 cDNA or control vector. Bars: 20μm. (D) Quantification of Ki-67 fluorescence intensity in BT549 cells expressing Ca_v_2.2 cDNA or control vector. P value was determined using Student’s t-test. Error bars represent mean ± s.d.. P value was determined using Student’s t-test (**p*<0.001). Error bars represent mean ± s.d.

**Supplementary Figure 4**

Ca_v_2.2 expression were determined by qRT-PCR in MDA-MB-231 cells stably expressing Ca_v_2.2 shRNA or control shRNA. P value was determined using Student’s t-test (**p*<0.001). Error bars represent mean ± s.d.

**Supplementary Figure 5**

Ca_v_2.2 expression were determined by qRT-PCR in MDA-MB-436 cells stably expressing Ca_v_2.2 shRNA or control shRNA. P value was determined using Student’s t-test (**p*<0.001). Error bars represent mean ± s.d.

**Supplementary Figure 6**

Human breast cancer MDA-MB-231 cells expressing Ca_v_2.2 shRNA or control shRNA were subjected to (A) CCK8 assay, (B) cell counting assay, (C) Ki-67 cell proliferation assay. The protein expression of Ki-67 was determined by immunofluorescence in MDA-MB-231 cells stably expressing Ca_v_2.2 shRNA or control shRNA. Bars: 20μm. (D) Quantification of Ki-67 fluorescence intensity in MDA-MB-231 cells stably expressing Ca_v_2.2 shRNA or control shRNA. P value was determined using Student’s t-test. Error bars represent mean ± s.d.. P value was determined using Student’s t-test (**p*<0.001). Error bars represent mean ± s.d.

**Supplementary Figure 7**

Human breast cancer MDA-MB-436 cells expressing Ca_v_2.2 shRNA or control shRNA were subjected to (A) CCK8 assay, (B) cell counting assay, (C) Ki-67 cell proliferation assay. The protein expression of Ki-67 was determined by immunofluorescence in MDA-MB-436 cells stably expressing Ca_v_2.2 shRNA or control shRNA. Bars: 20μm. (D) Quantification of Ki-67 fluorescence intensity in MDA-MB-436 cells stably expressing Ca_v_2.2 shRNA or control shRNA. P value was determined using Student’s t-test. Error bars represent mean ± s.d.. P value was determined using Student’s t-test (**p*<0.001). Error bars represent mean ± s.d.

**Supplementary Figure 8**

(**A**) Quantification of cortactin fluorescence intensity in MDA-MB-231 cells stably expressing Ca_v_2.2 shRNA or control shRNA cells. P value was determined using Student’s t-test (**p*<0.001). Error bars represent mean ± s.d. (**B**) Quantification of cortactin fluorescence intensity in BT-549 cells transfected with Ca_v_2.2 cDNA or a control vector. P value was determined using Student’s t-test (**p*<0.05).

**Supplementary Figure 9**

(**A**) Cortactin expression were determined by immunoblotting in MDA-MB-231 cells expressing USP43 cDNA, or OTUD1 cDNA, or OTUD4 cDNA, or OTUD7A cDNA, USP40 cDNA, or USP54 cDNA, or control vector. The expression of USP43 (**B**), OTUD1 (**C**), OTUD4 (**D**), OTUD7A (**E**), USP40 (**F**), USP54 (**G**), were determined by qRT-PCR in MDA-MB-231 cells expressing the cDNA of these genes respectively.

**Supplementary Figure 10**

(**A**)[Ca^2+^]i was measured by calcium sensitive dye Fura-2AM in MDA-MB-231 cells expressing Ca_v_2.2 shRNA or a control shRNA. (**B**) [Ca^2+^]i was measured by calcium sensitive dye Fura-2AM in BT-549 cells expressing Ca_v_2.2 cDNA or a control vector.

**Supplementary Figure 11**

(**A**) USP43 expression were determined by qRT-PCR in MDA-MB-231 cells expressing NFAT3 shRNA or a control shRNA. Error bars represent mean ± s.d. P value was determined using Student’s t-test (**p*<0.001). (**B**) expression NFAT3 expression was determined by qRT-PCR in MDA-MB-231 cells expressing NFAT3 shRNA or a control shRNA. P value was determined using Student’s t-test (**p*<0.001). Error bars represent mean ± s.d.

**Supplementary Figure 12**

(**A**) USP43 expression were determined by qRT-PCR in MDA-MB-231 cells expressing NFAT1 shRNA, or NFAT4 shRNA, or a control shRNA. The expression of NFAT1 (**B**), NFAT4 (**C**) were determined by qRT-PCR in MDA-MB-231 cells expressing the specific shRNA of these two genes respectively. P value was determined using Student’s t-test (**p*<0.001). Error bars represent mean ± s.d.

**Supplementary Figure 13**

NFAT3 and USP43 expressions were determined by immunoblotting in MDA-MB-231 cells expressing NFAT3 shRNAs or a control shRNA.

**Supplementary Figure 14**

Chromatin immunoprecipitation (ChIP) was performed using an anti-NFAT3 antibody or a control IgG. (A) The USP43 promoter region which contains consensus NFAT3 binding sequences did not show significant enrichment after immunoprecipitation by an anti-NFAT3 antibody. (B) The BACE1 promoter region where NFAT3 was previously validated to bind showed significant enrichment after immunoprecipitation by anti-NFAT3 antibody. Error bars represent mean ± s.d.

**Supplementary Figure 15**

Chromatin immunoprecipitation (ChIP) was performed using an anti-NFAT2 antibody or a control IgG. The BMI1 promoter region where NFAT2 was previously validated to bind showed significant enrichment after immunoprecipitation by an anti-NFAT2 antibody. Error bars represent mean ± s.d.
