# Supplementary material for: Cav2.2-NFAT2-USP43 axis promotes invadopodia formation and breast cancer metastasis through cortactin stabilization
Source: Cell Death Dis. 2022 Sep 22;13(9):812. doi: 10.1038/s41419-022-05174-0 (PMC9500045; doi:10.1038/s41419-022-05174-0)

Supplementary Figure 1

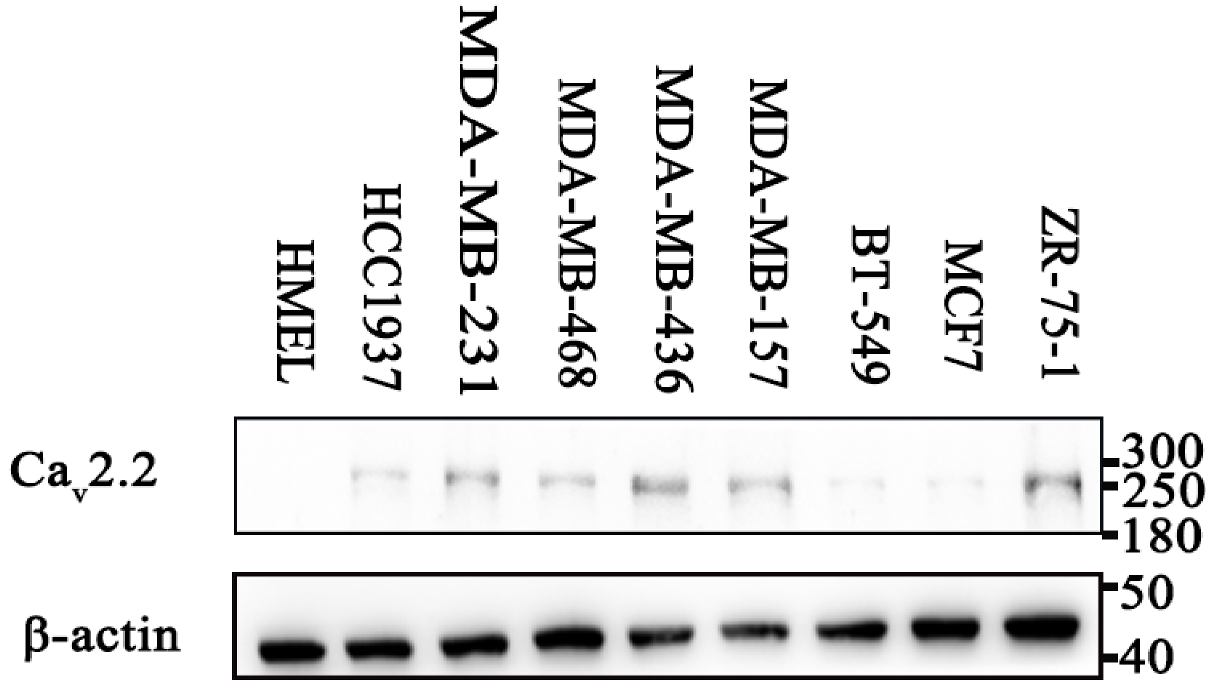

Supplementary Figure 2

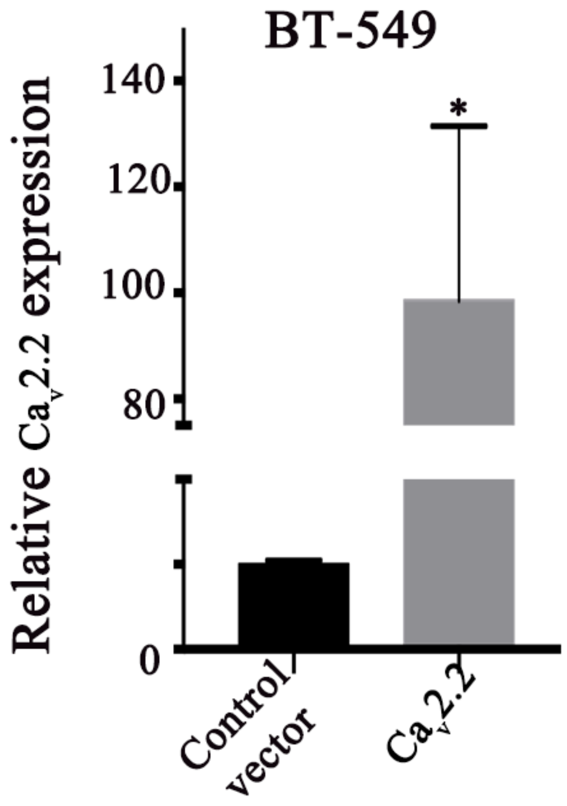

Supplementary Figure 3

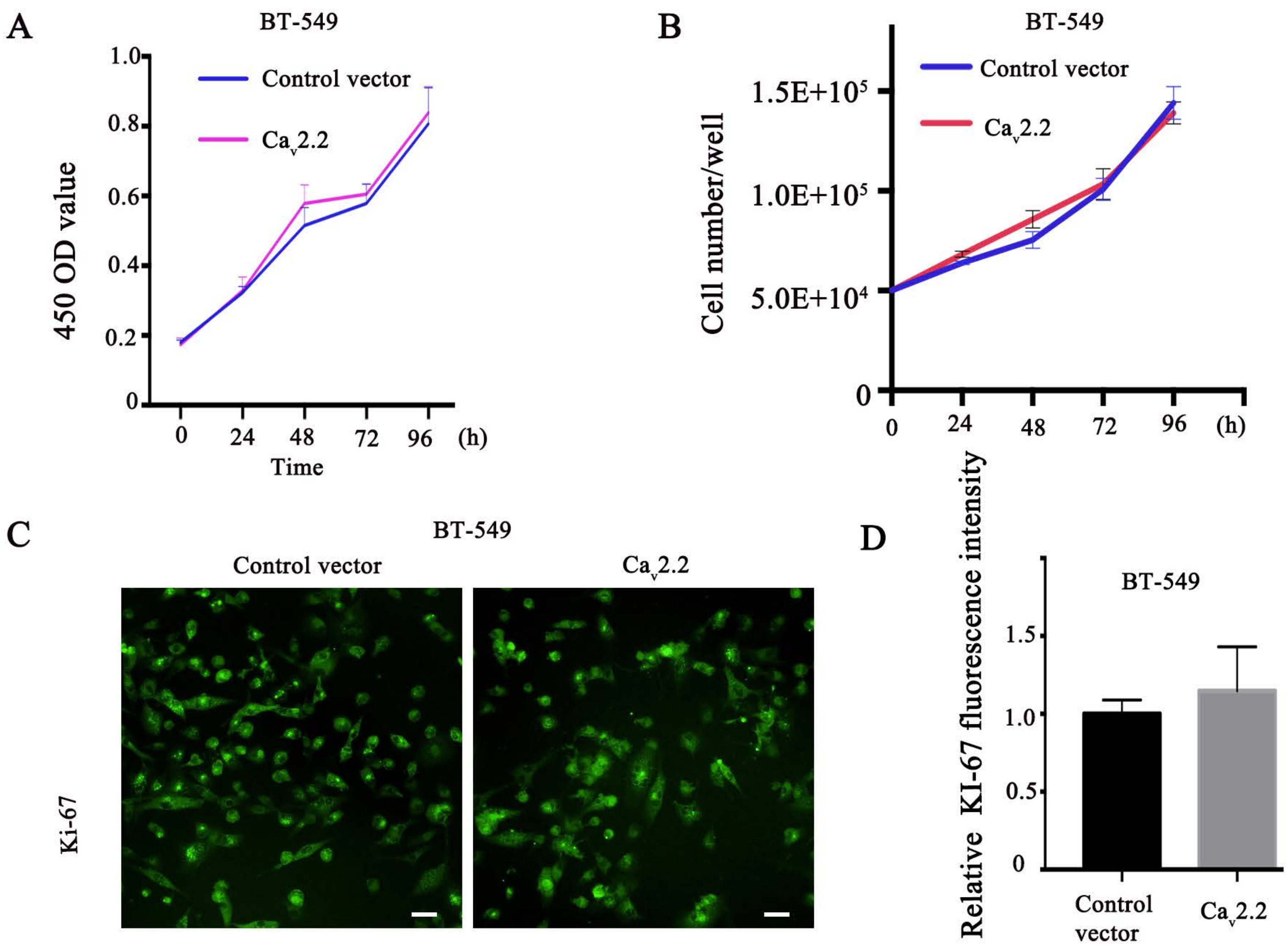

Supplementary Figure 4

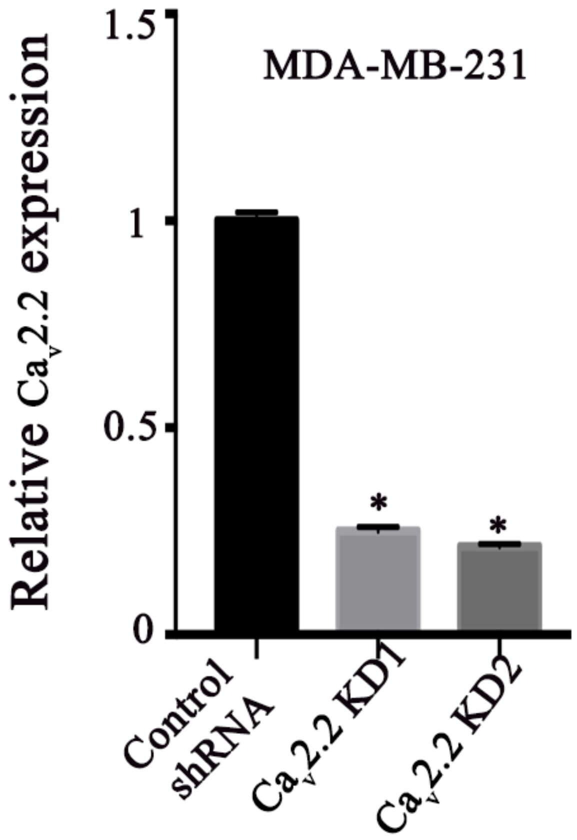

Supplementary Figure 5

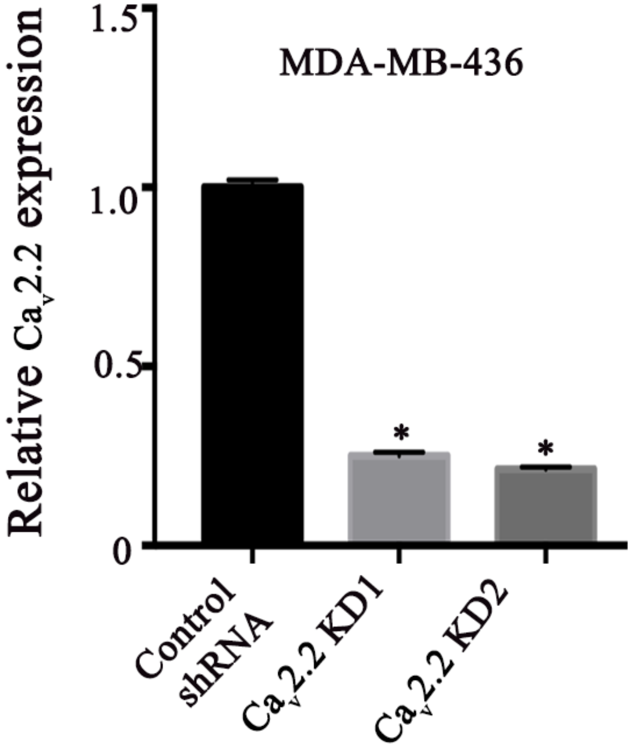

Supplementary Figure 6

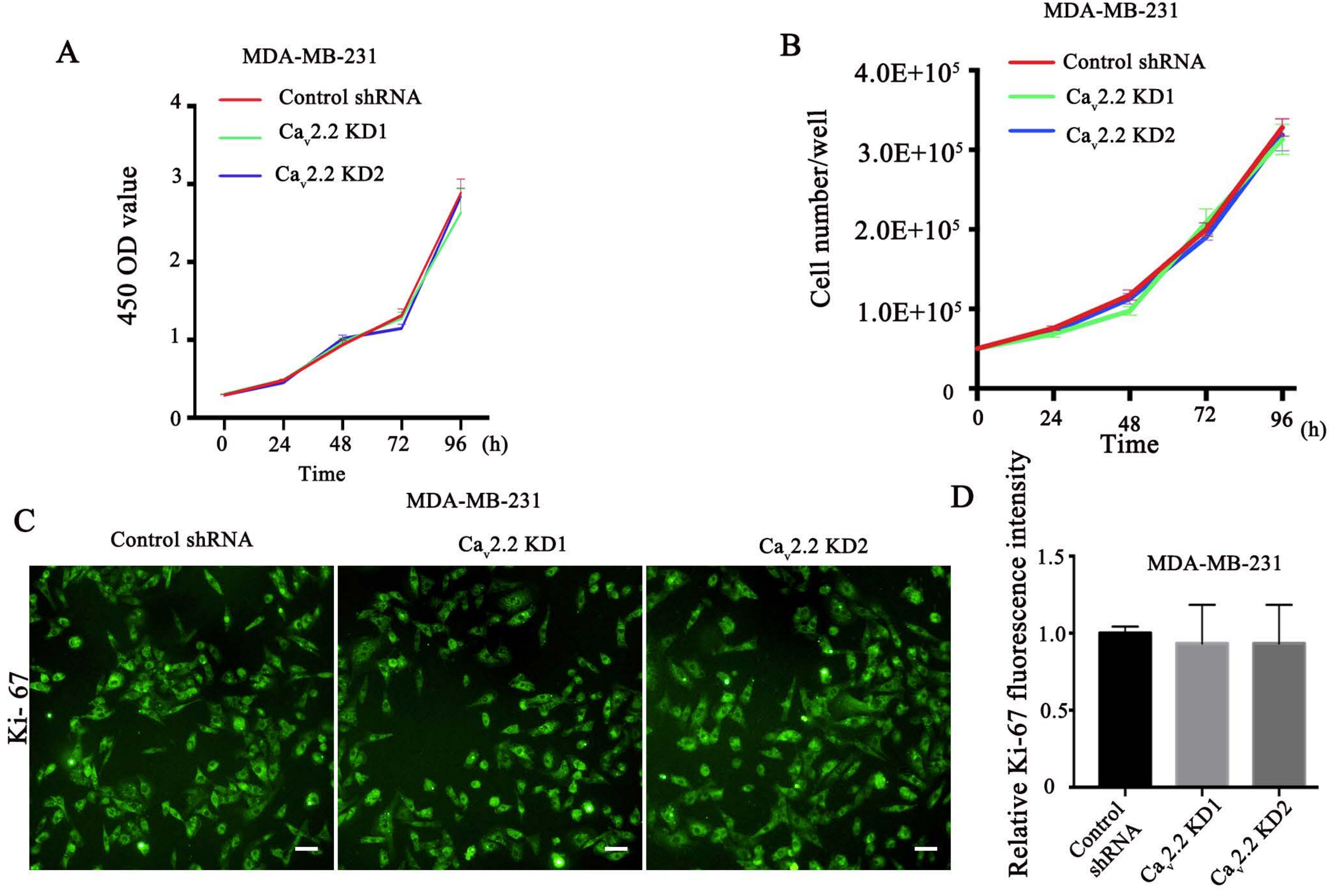

Supplementary Figure 7

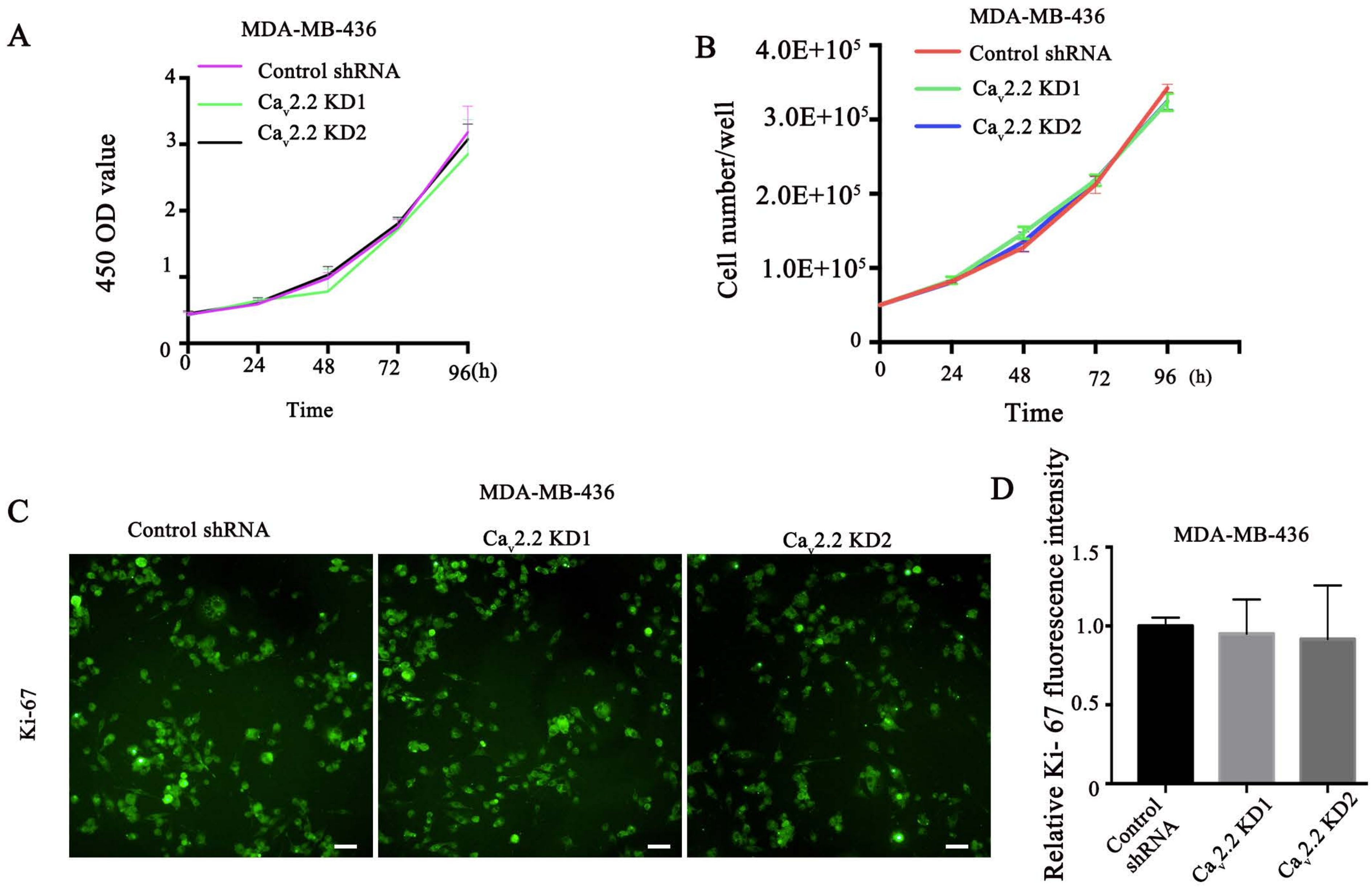

Supplementary Figure 8

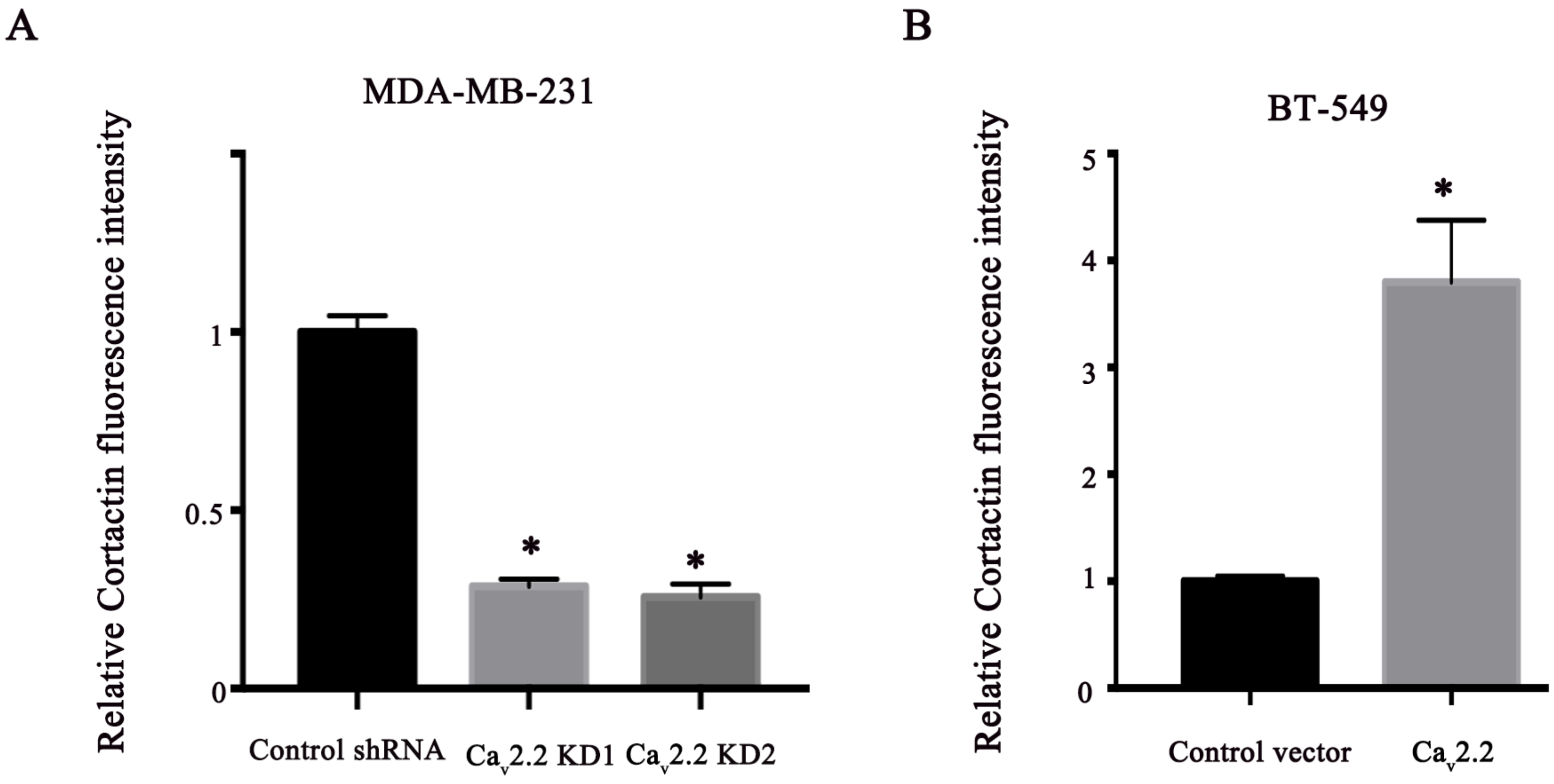

Supplementary Figure 9

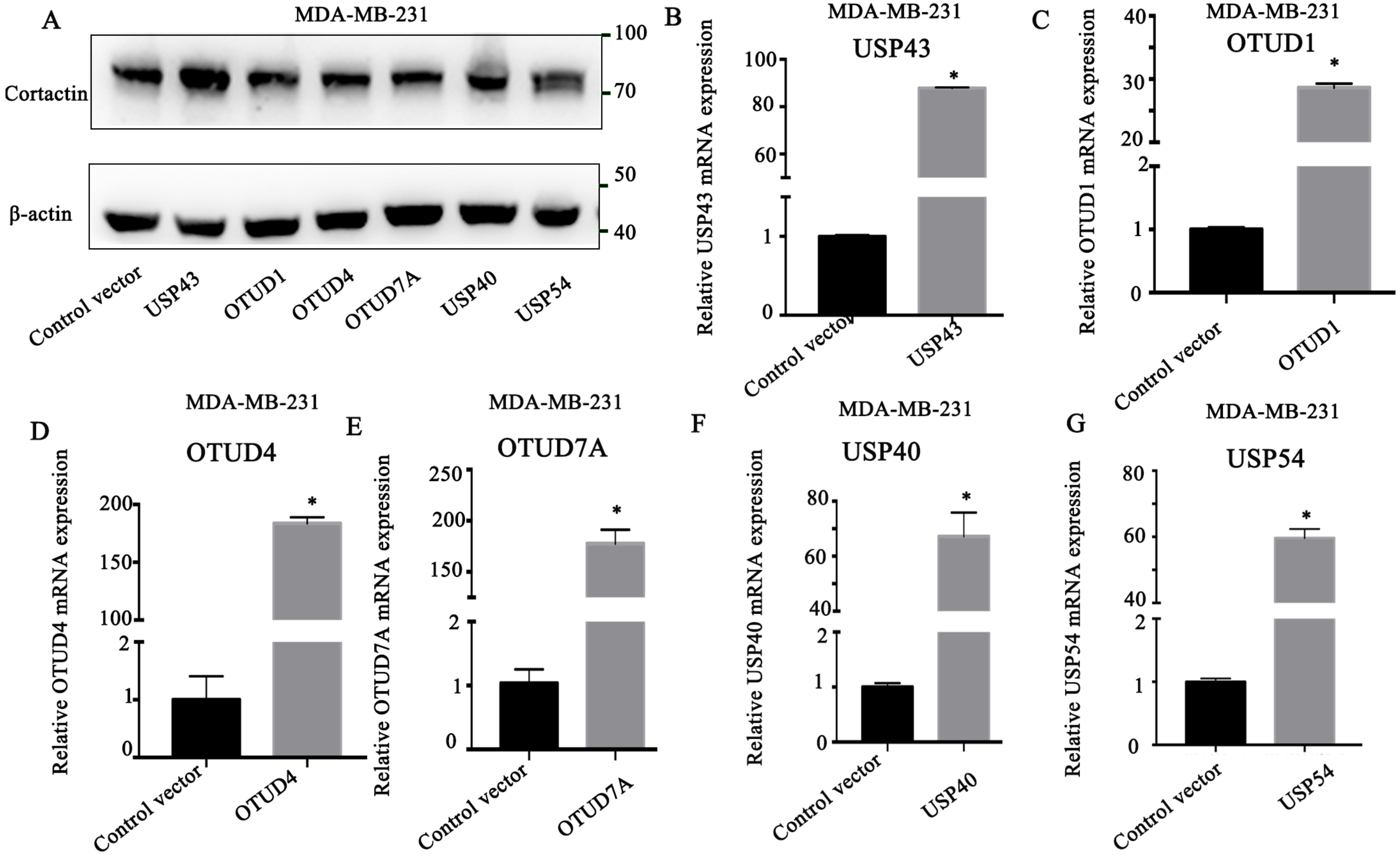

Supplementary Figure 10

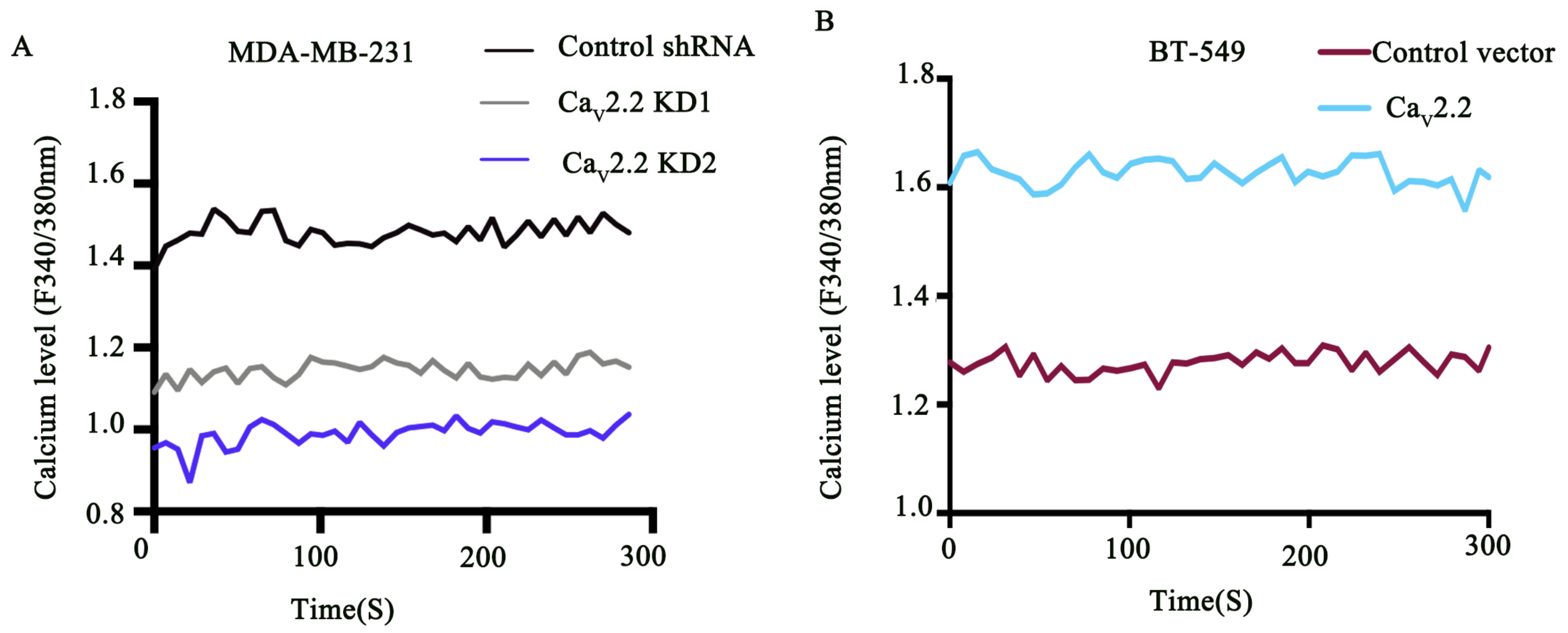

Supplementary Figure 11

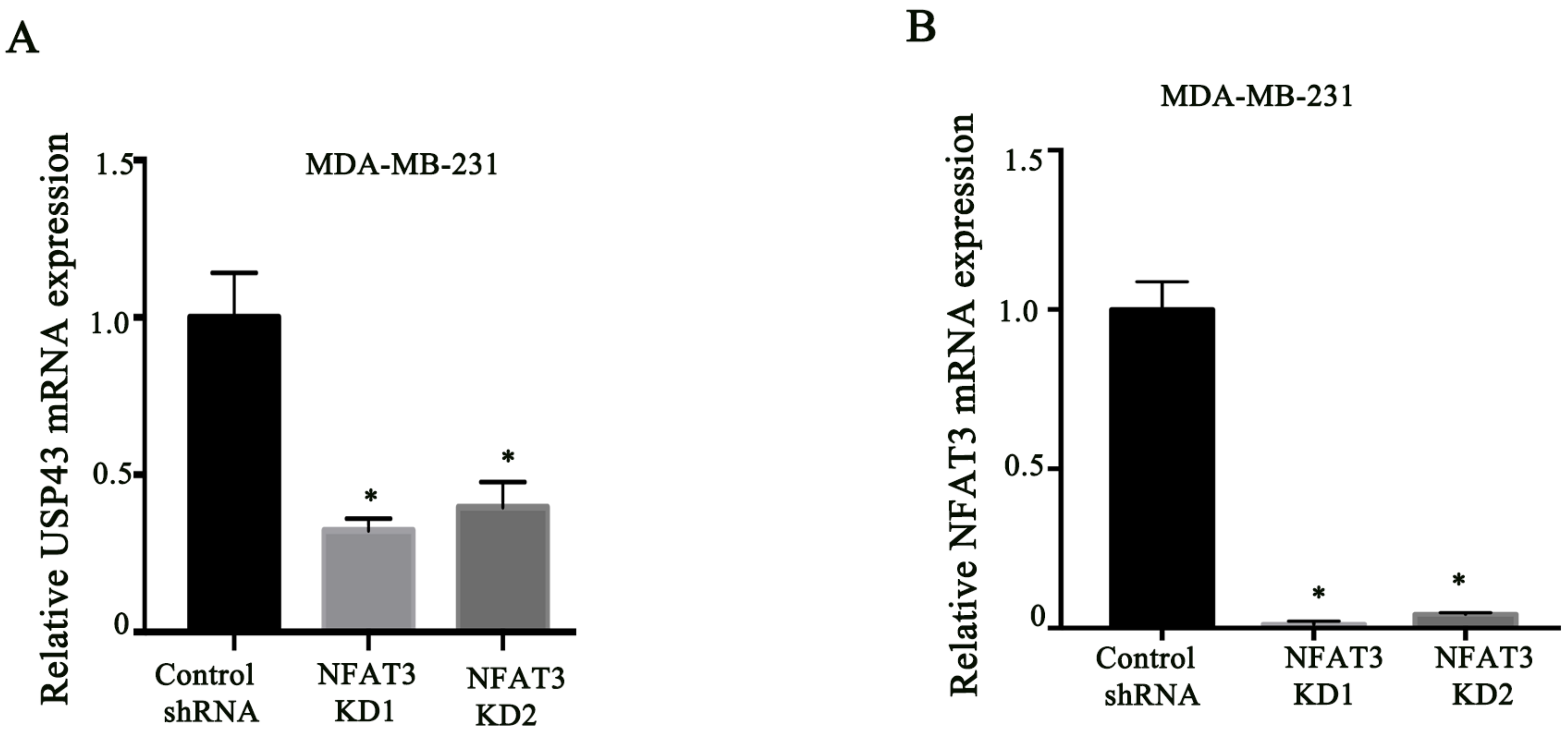

Supplementary Figure 12

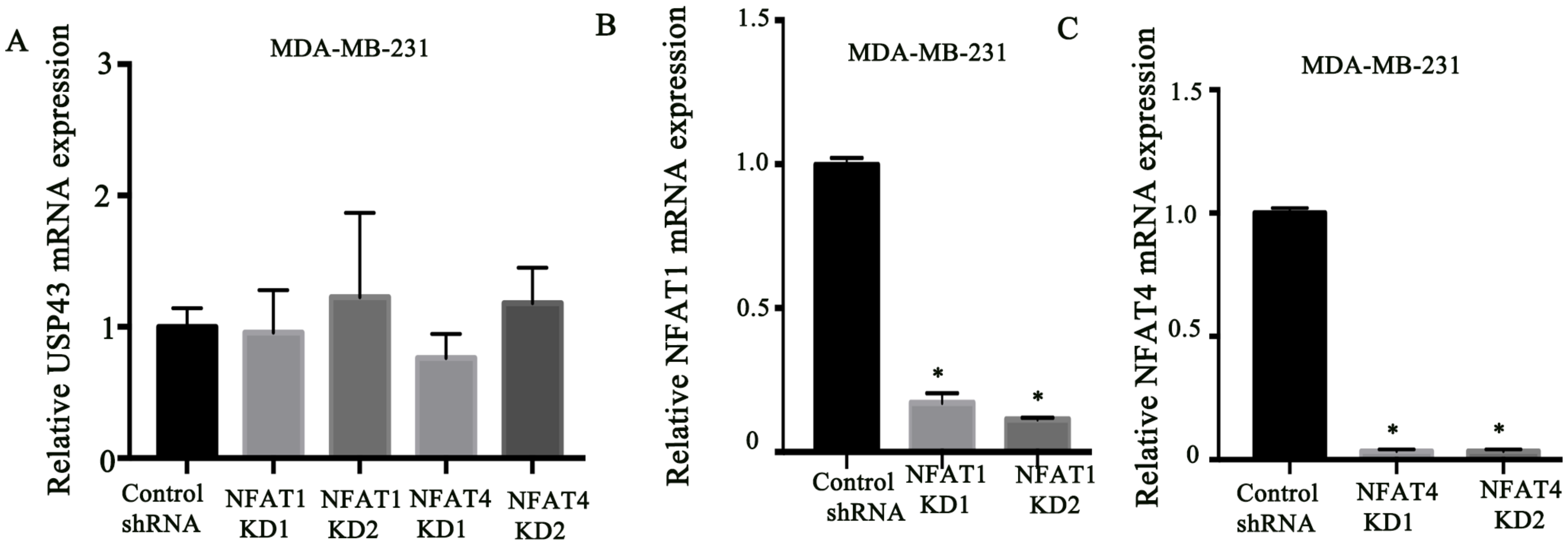

Supplementary Figure 13

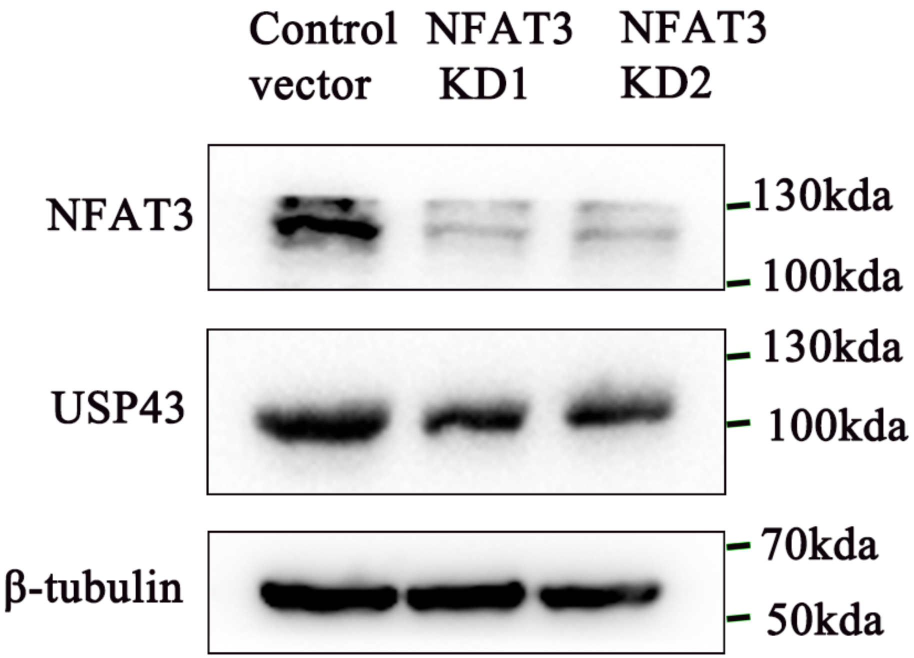

Supplementary Figure 14

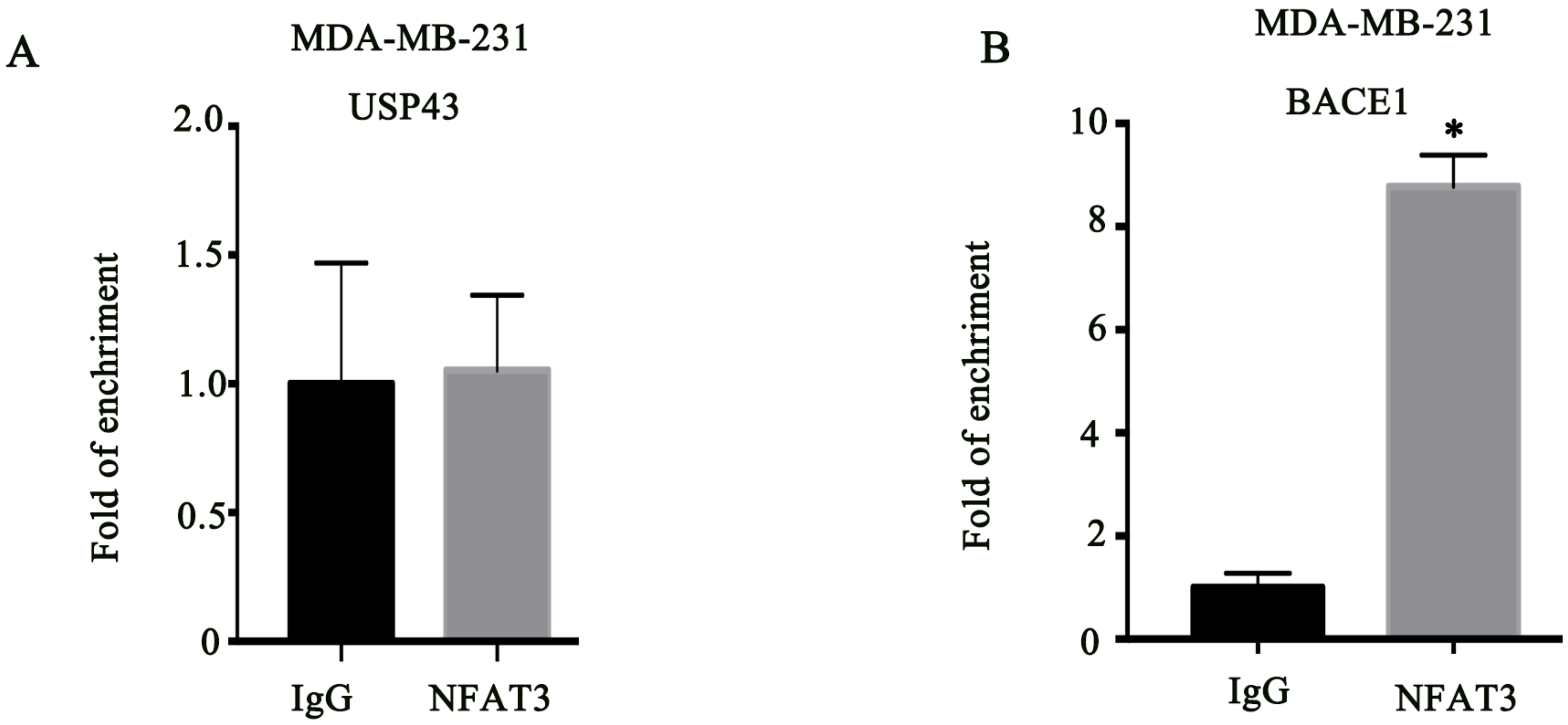

Supplementary Figure 15

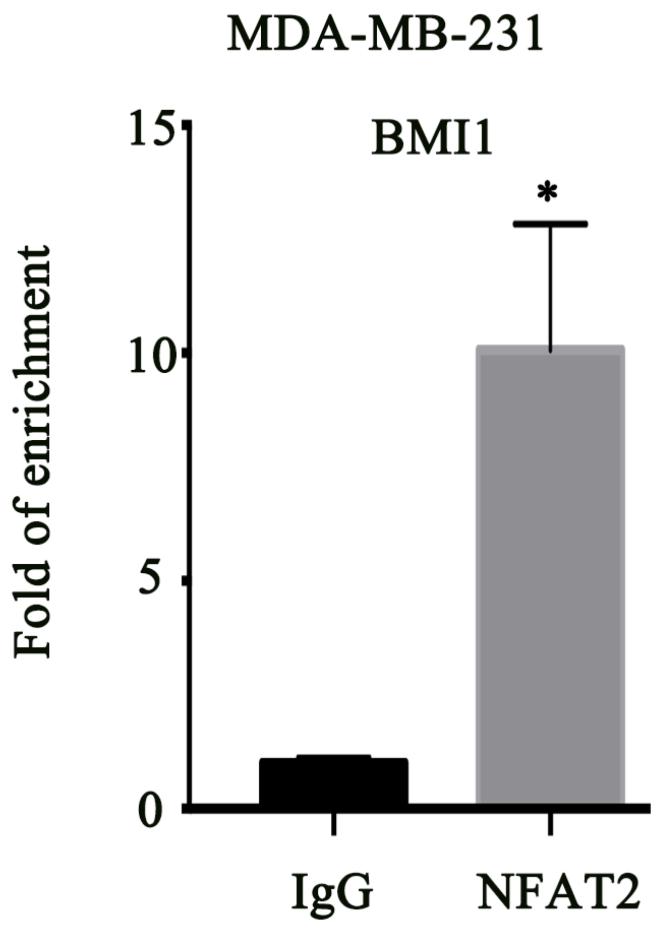

Figure 4A

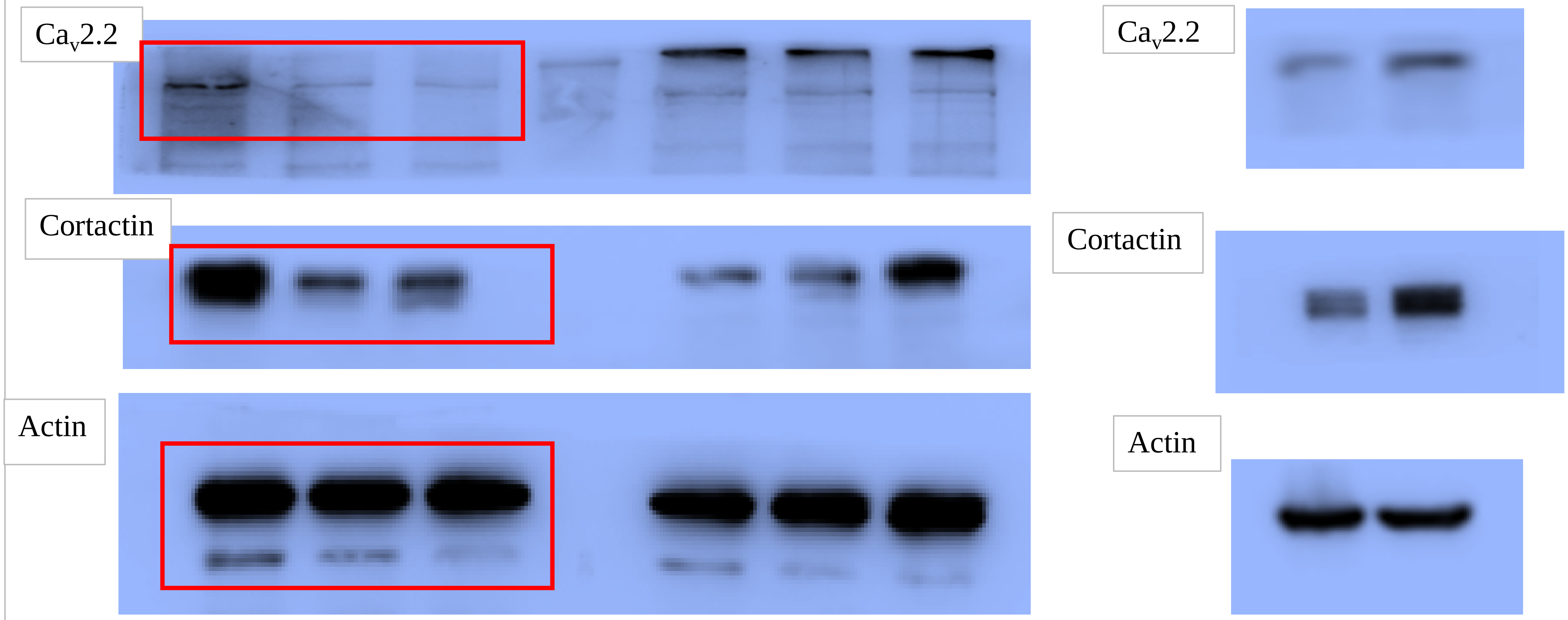

Figure 4D

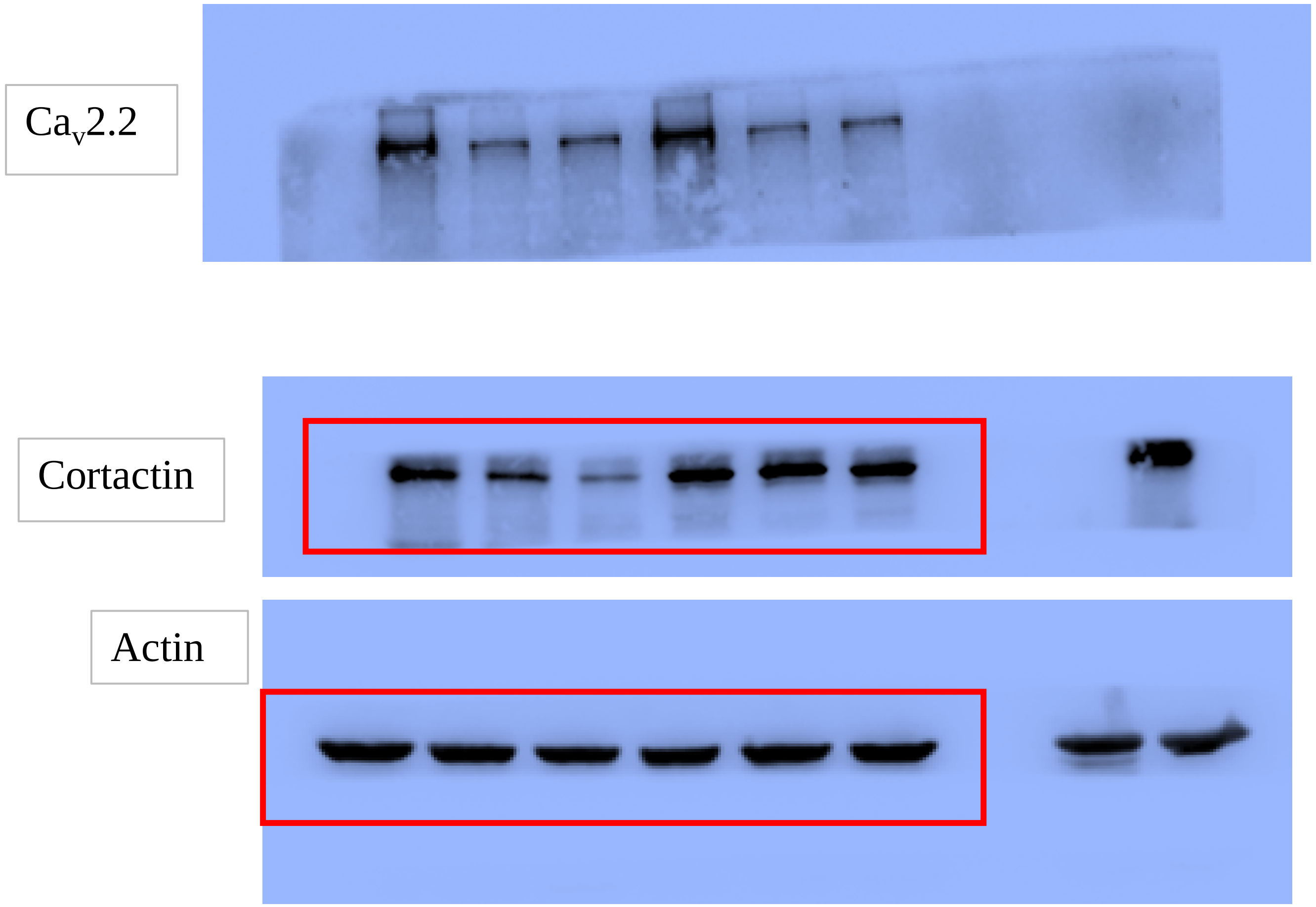

Figure 4E

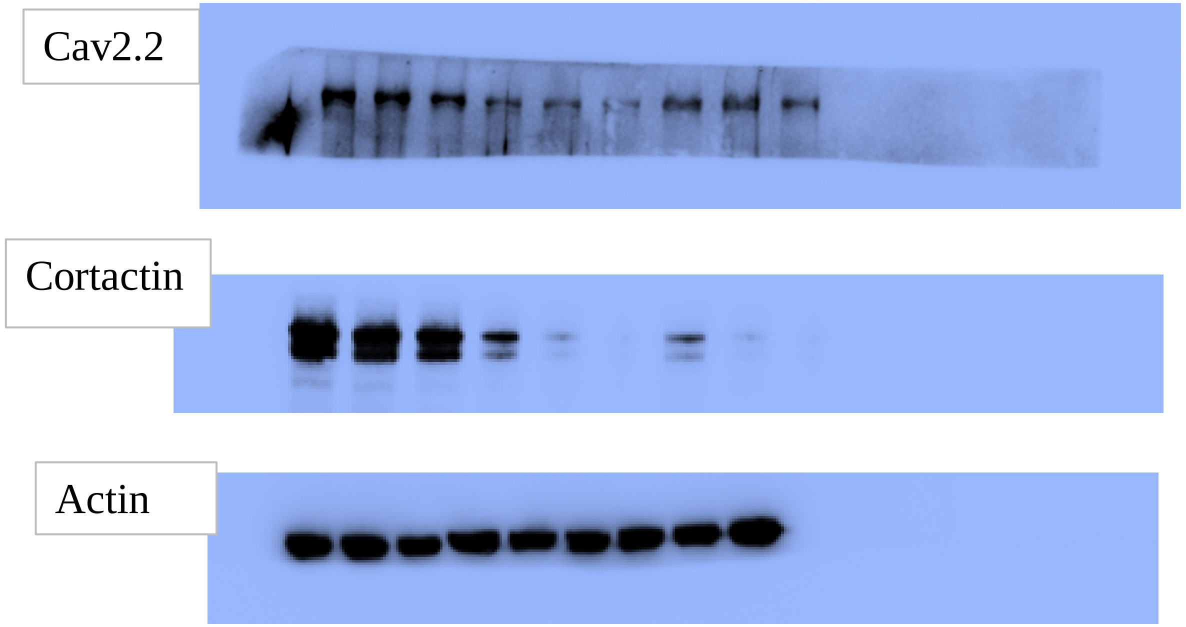

Figure 4F

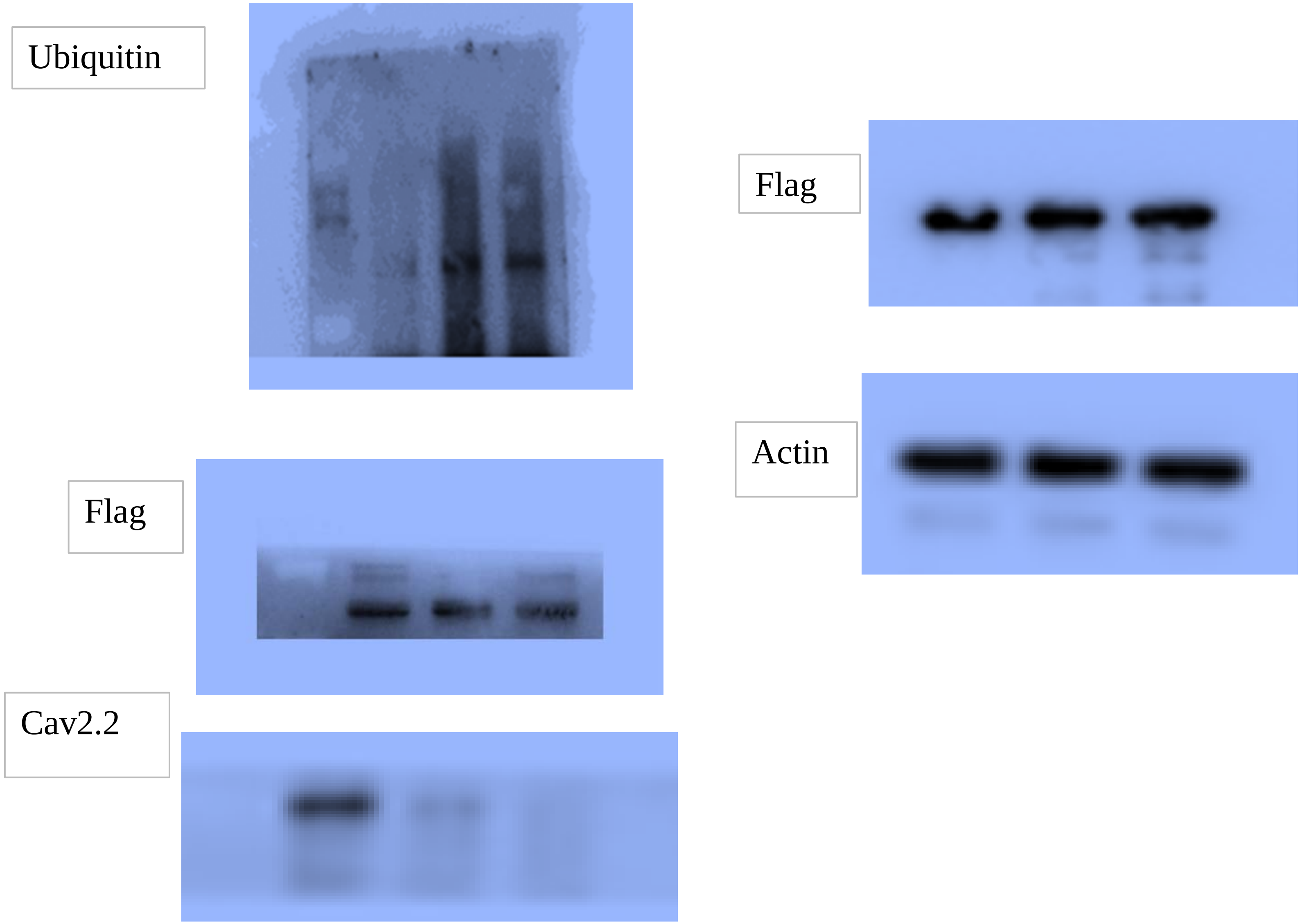

Figure 5A

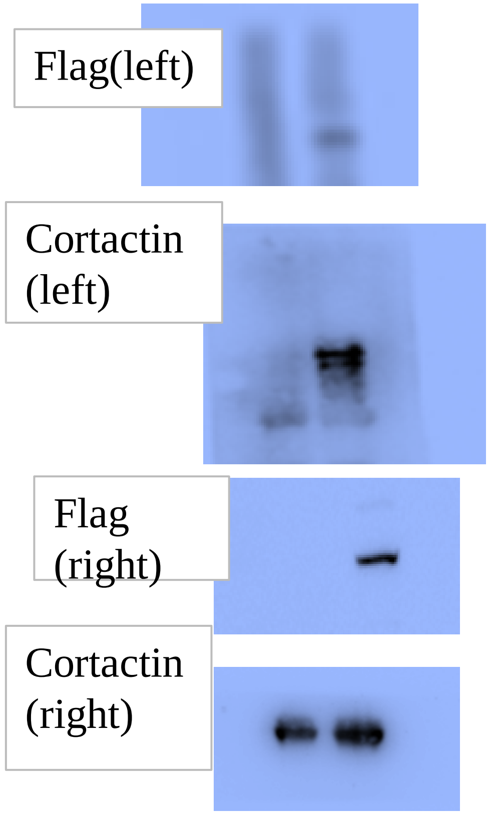

Figure 5B

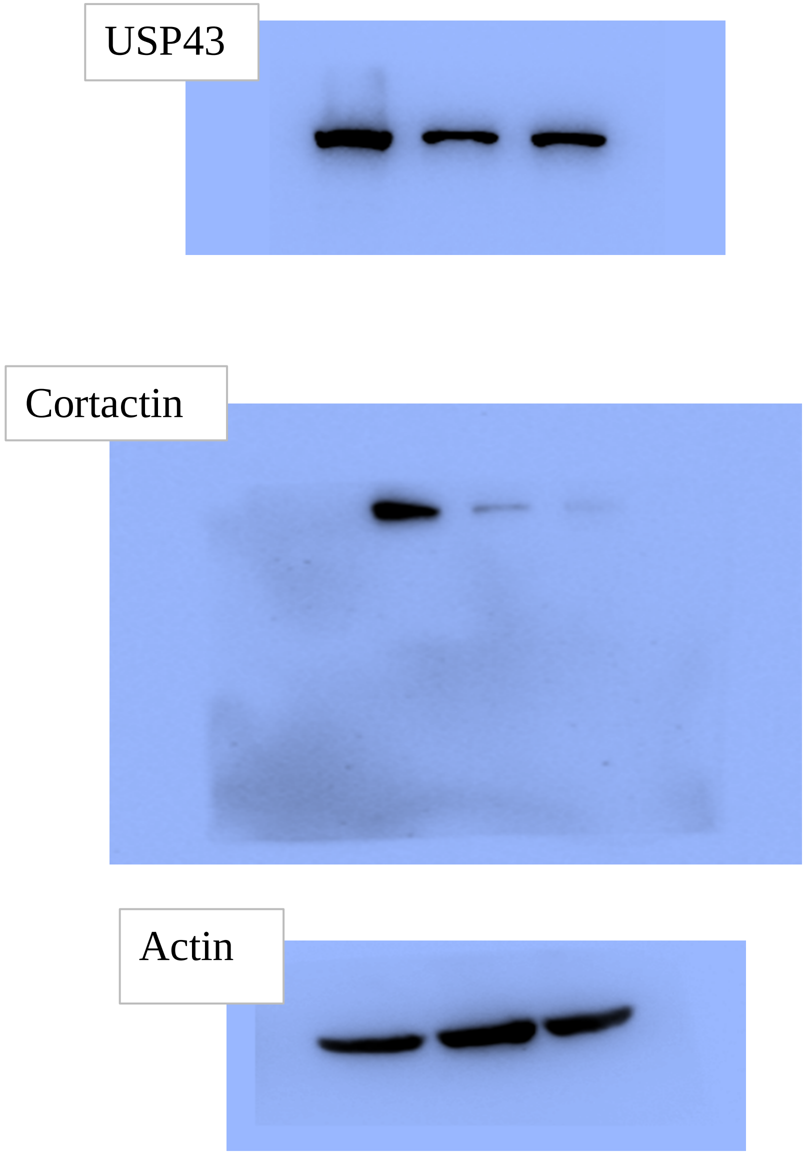

Figure 5D

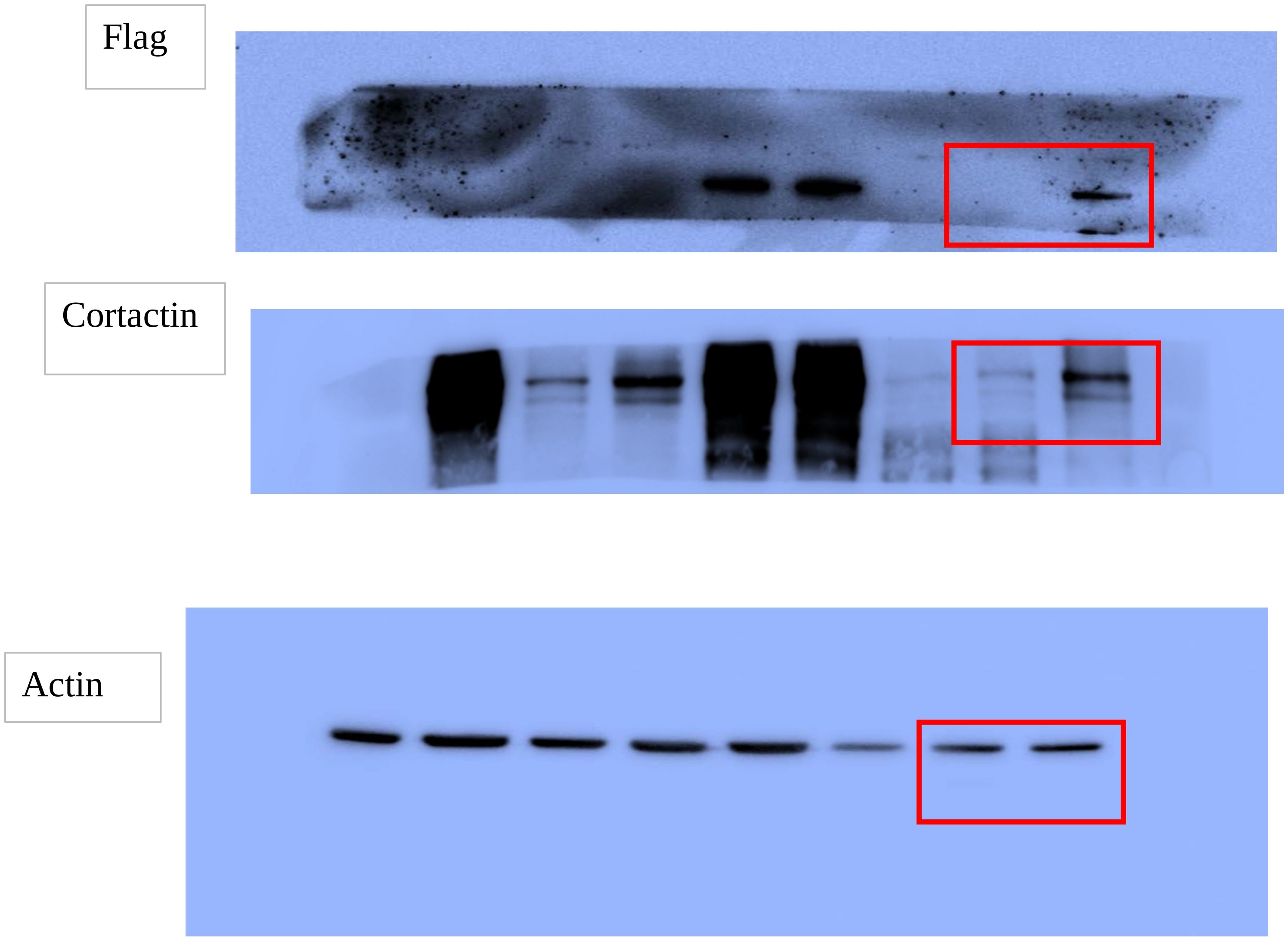

Figure 5F

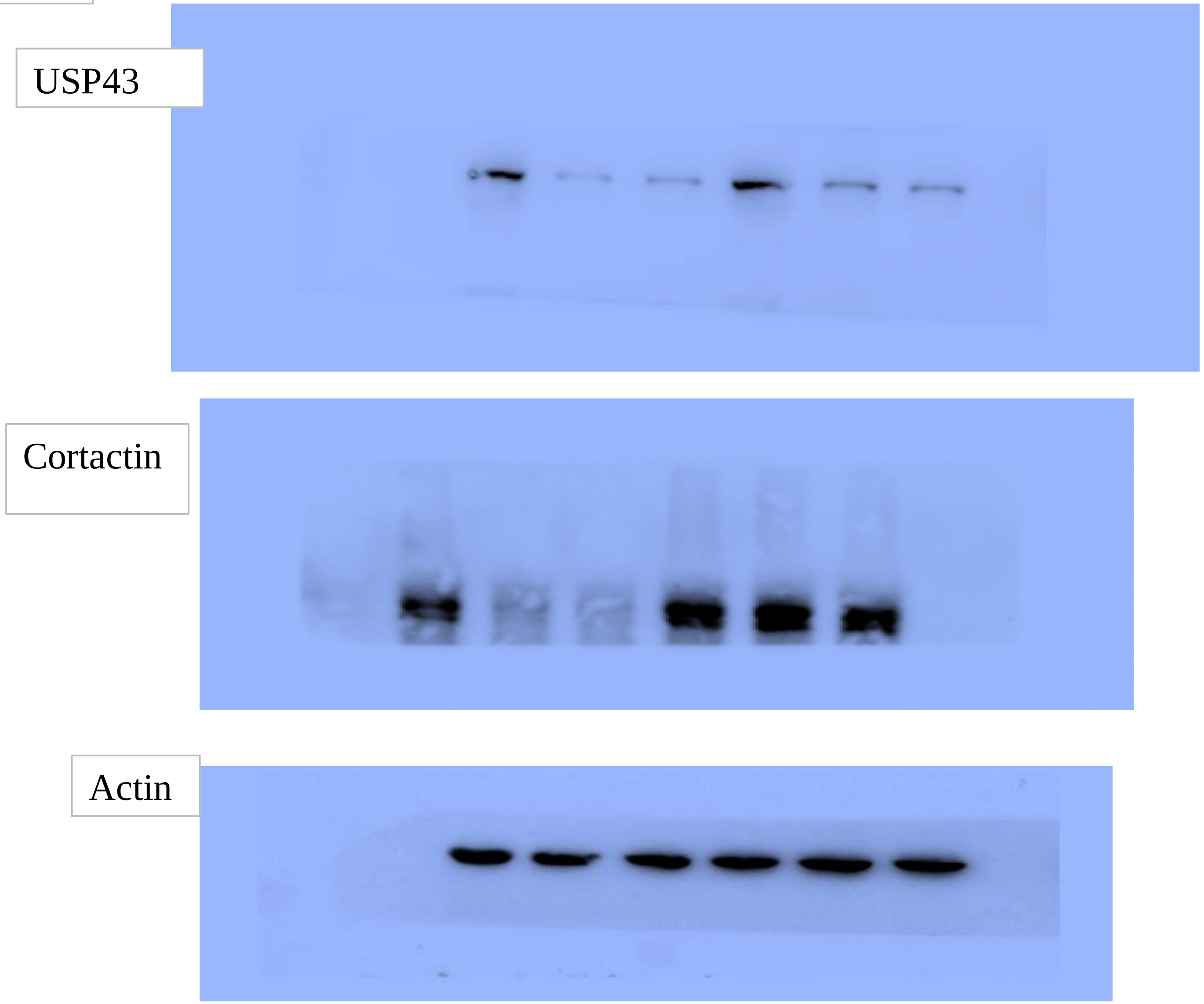

Figure 5G

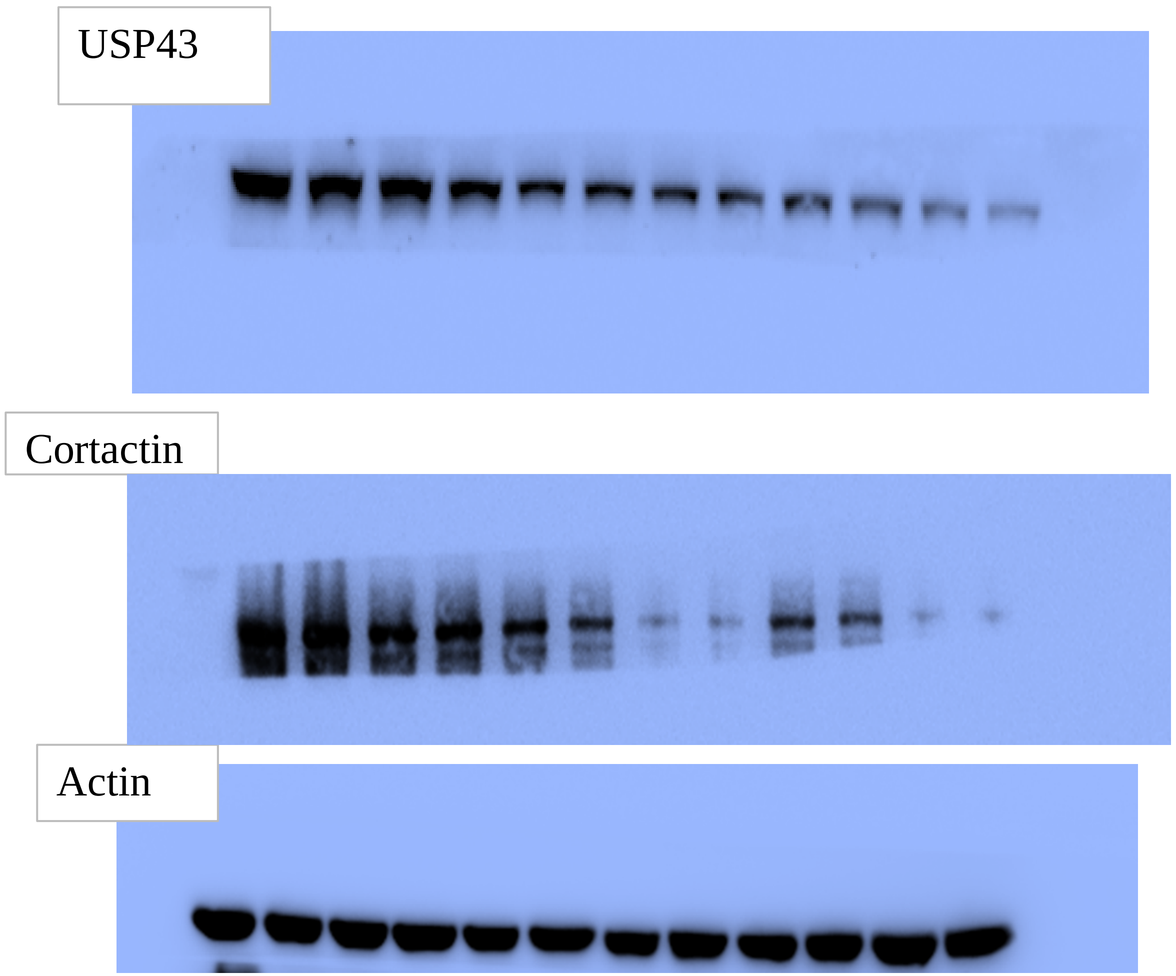

Figure 5H

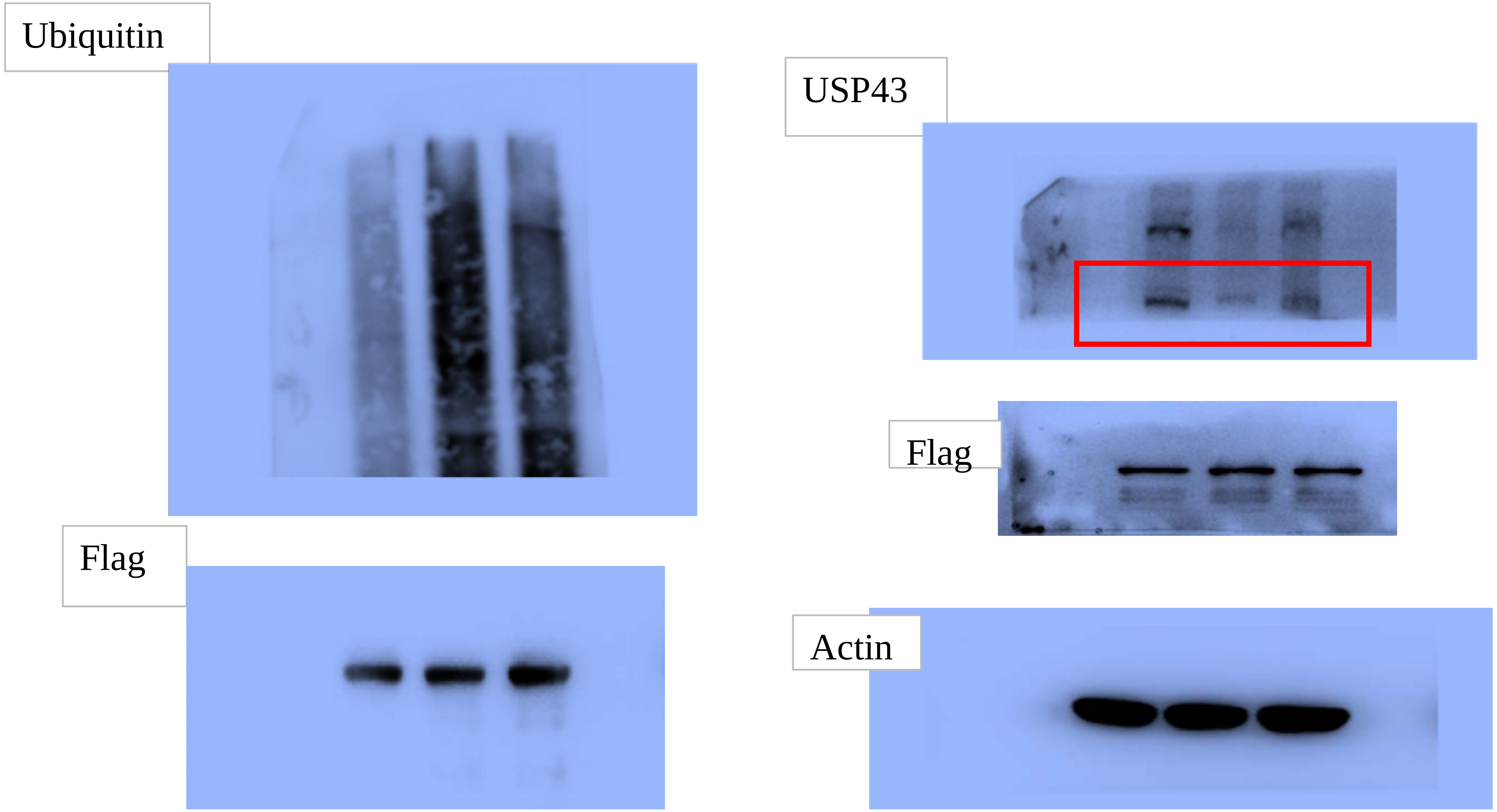

Figure 6A

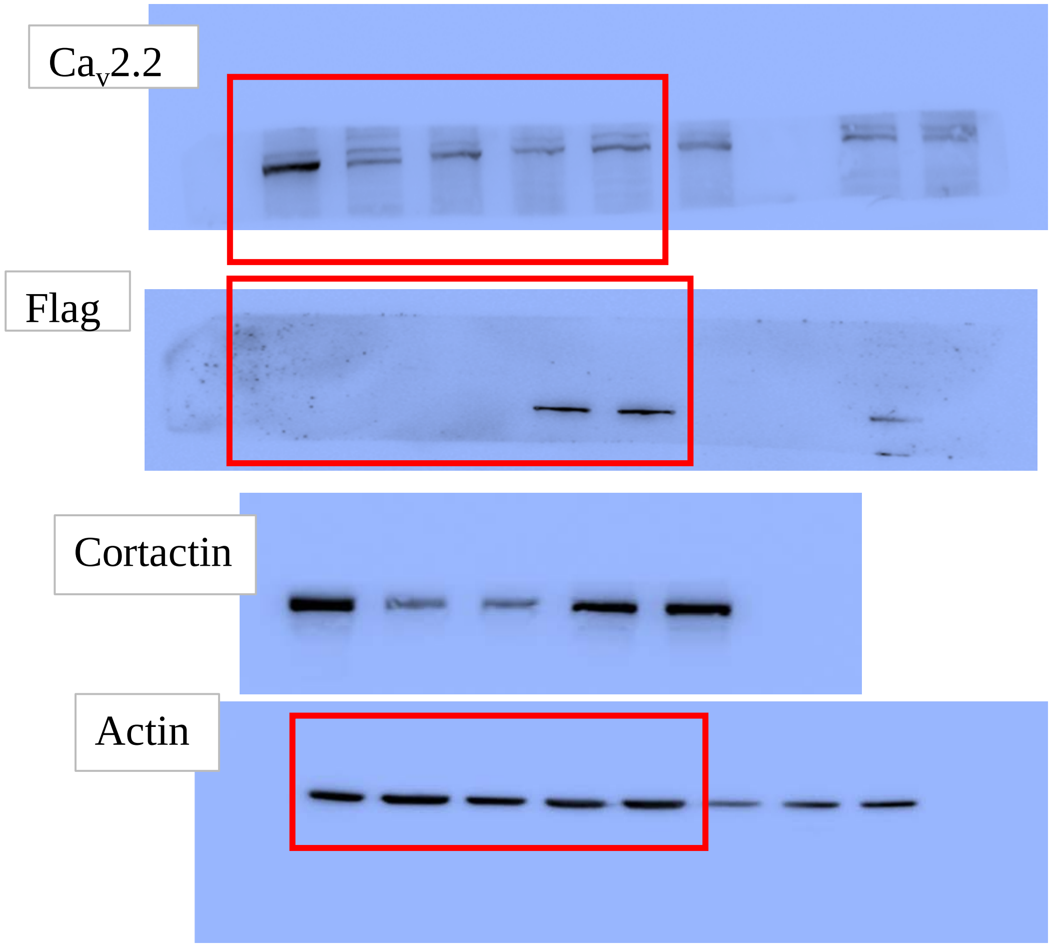

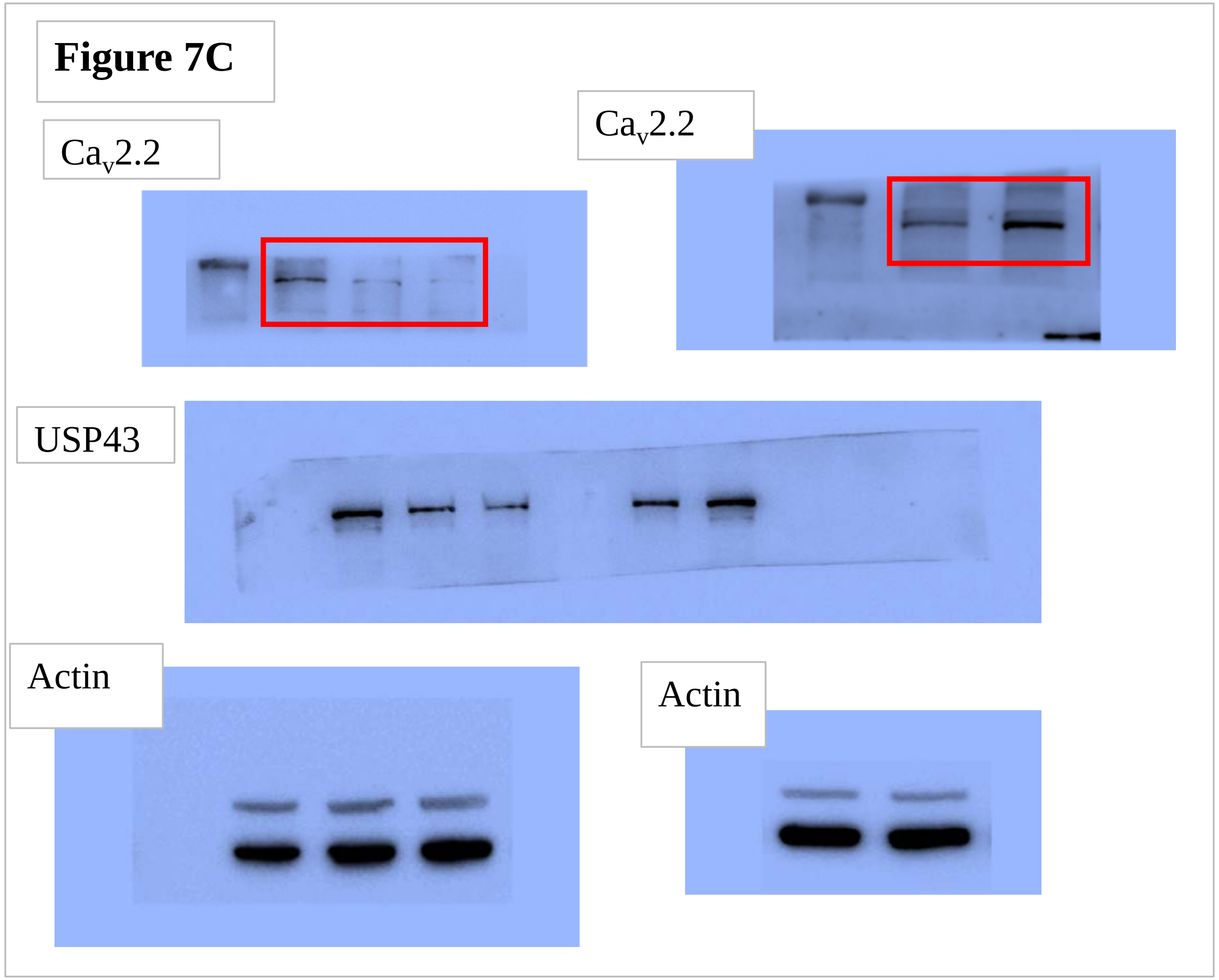
Figure 7C
Cav2.2
Cav2.2
USP43
Actin
Actin

Figure 7E

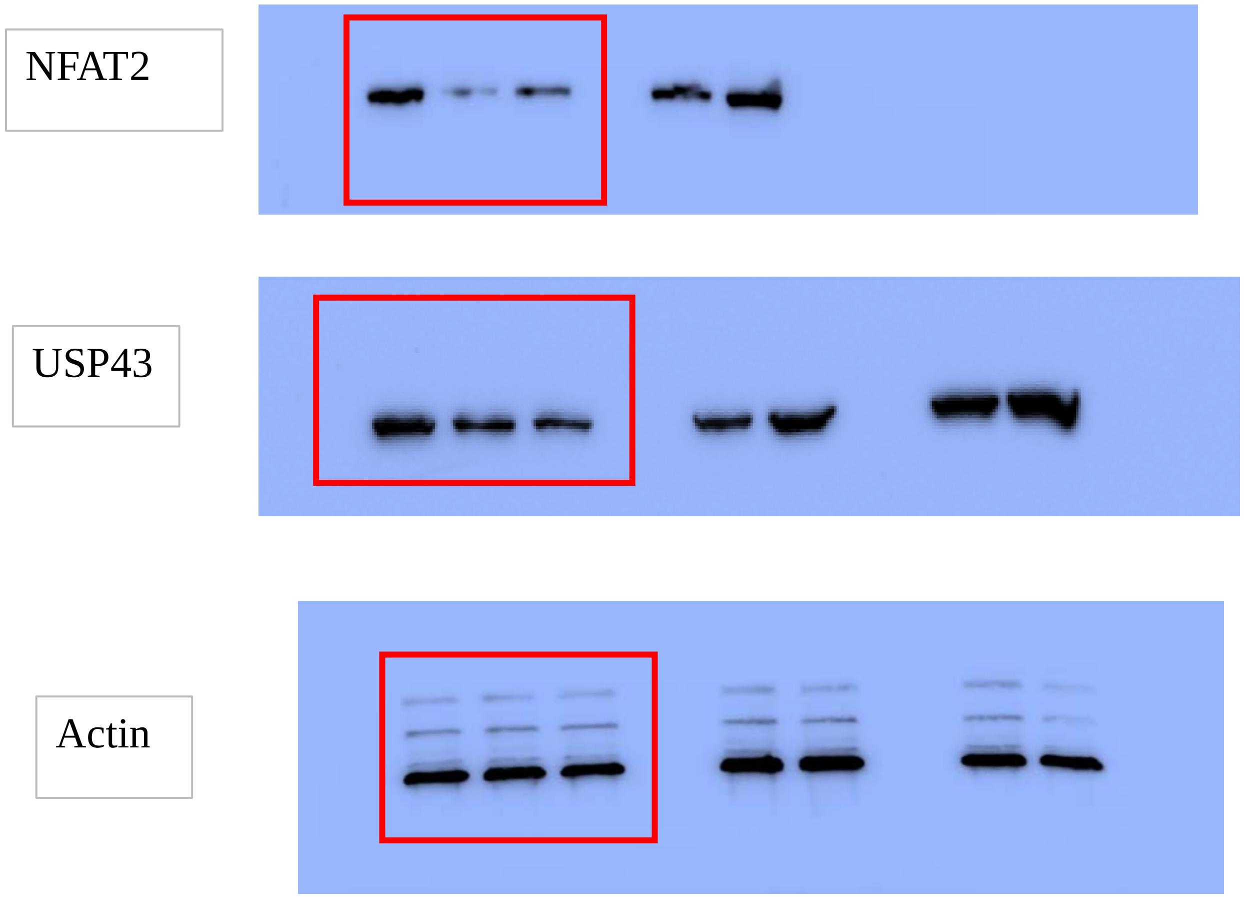

Figure 7G

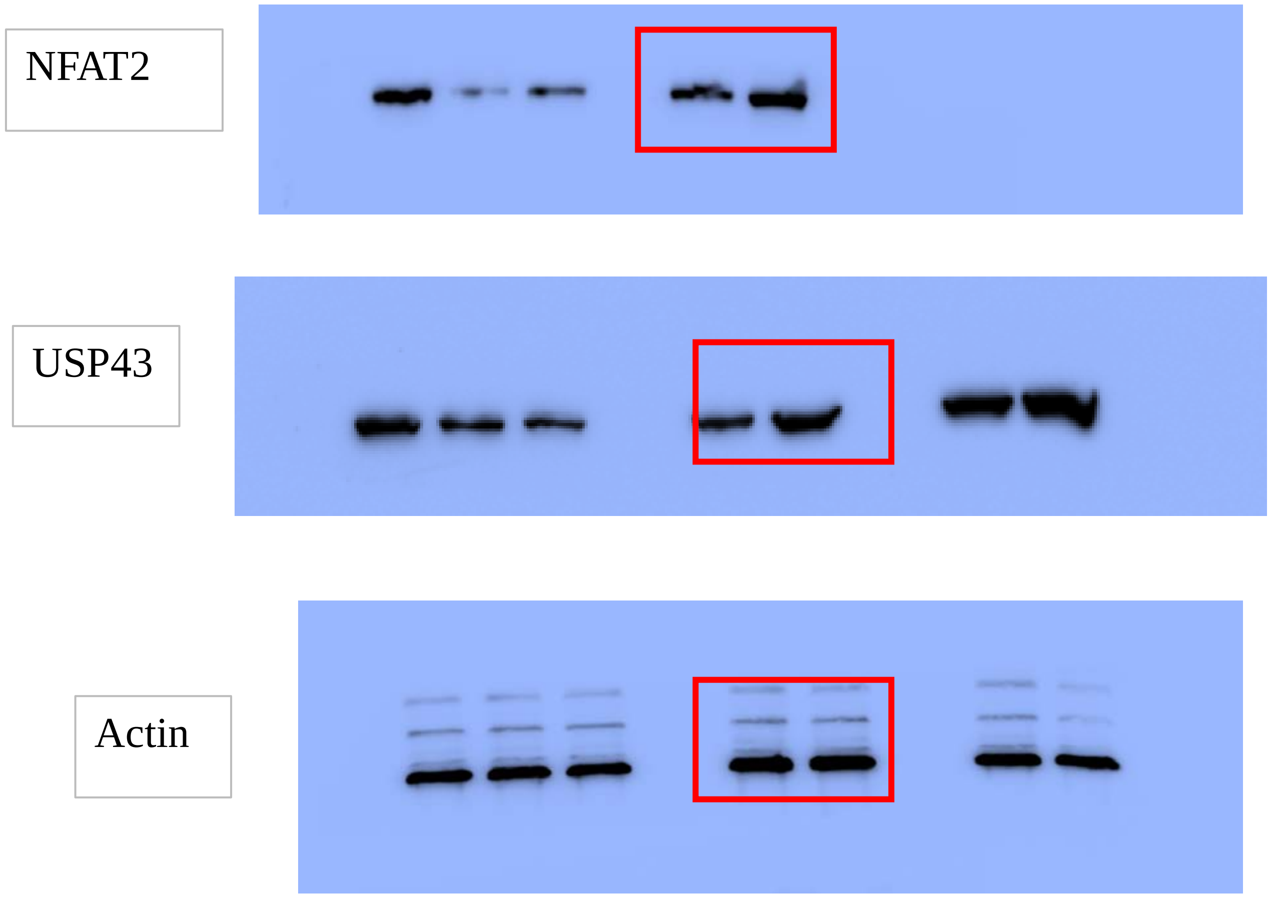

Figure 8B

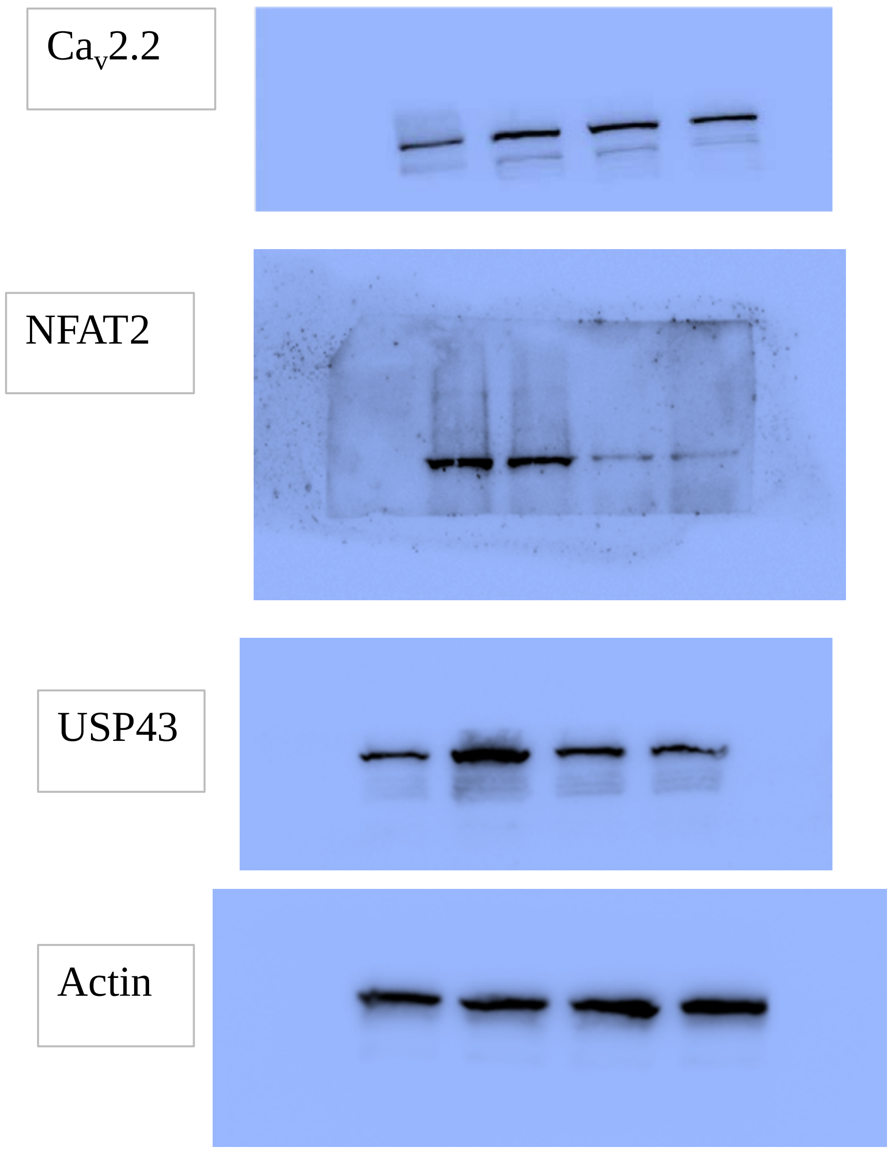

Figure 8C

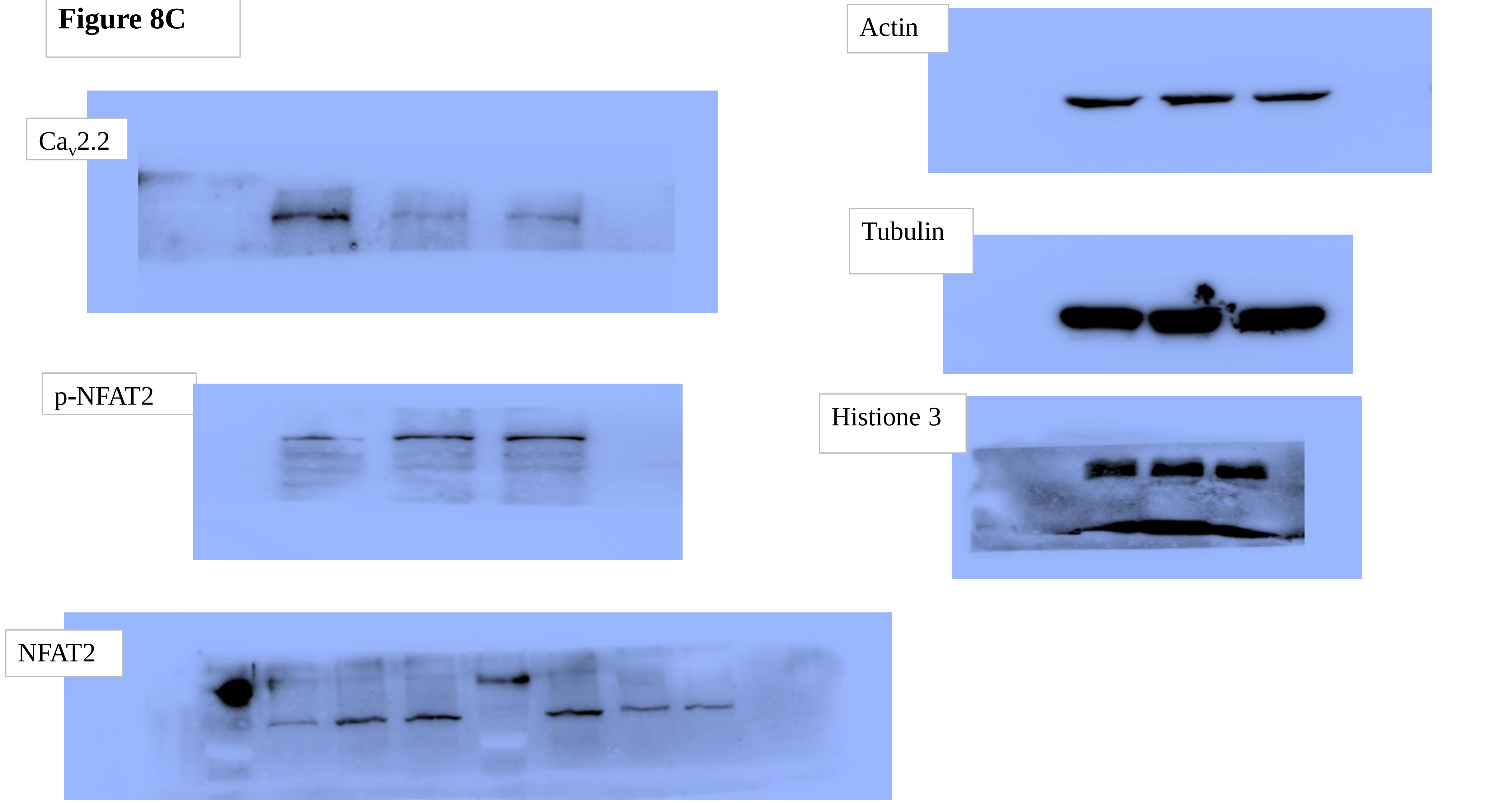

Supplementary Figure 1

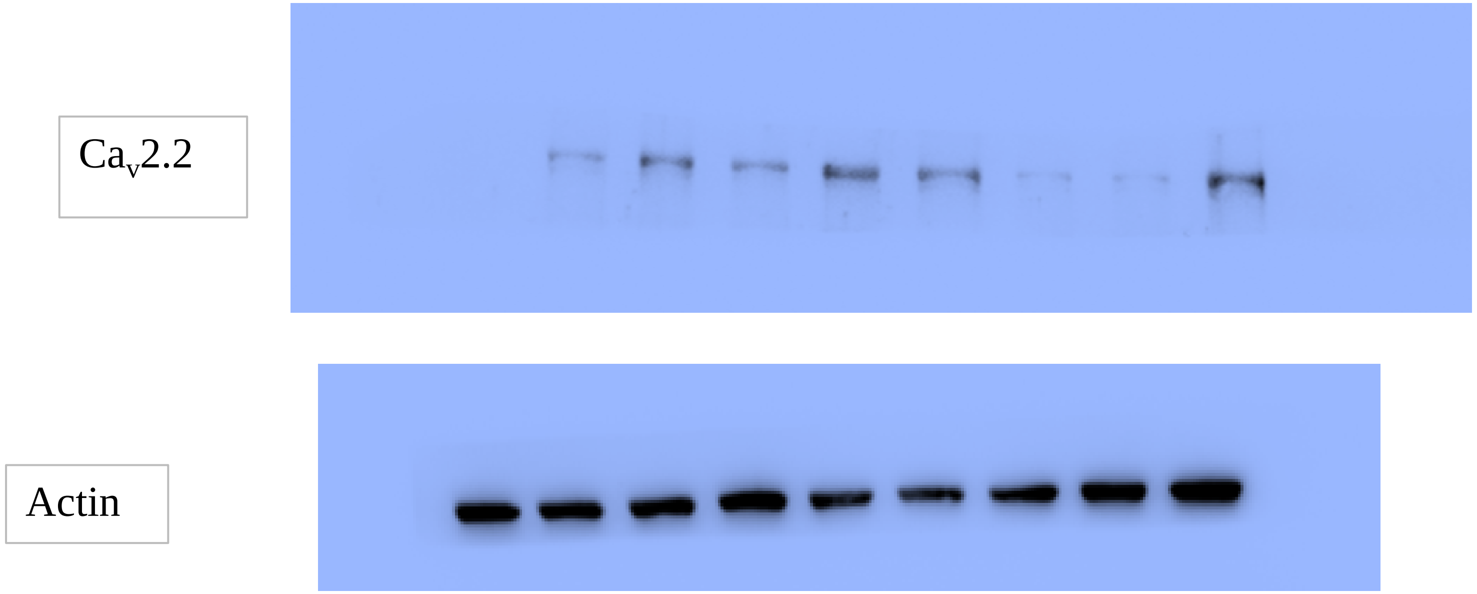

Supplementary Figure 9

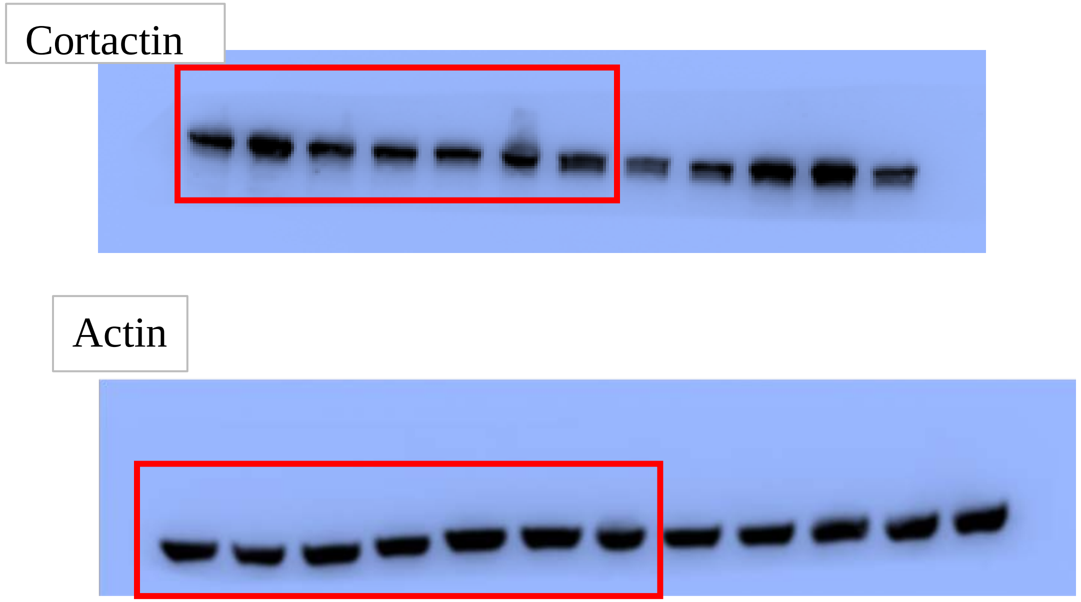

Supplementary Figure 13

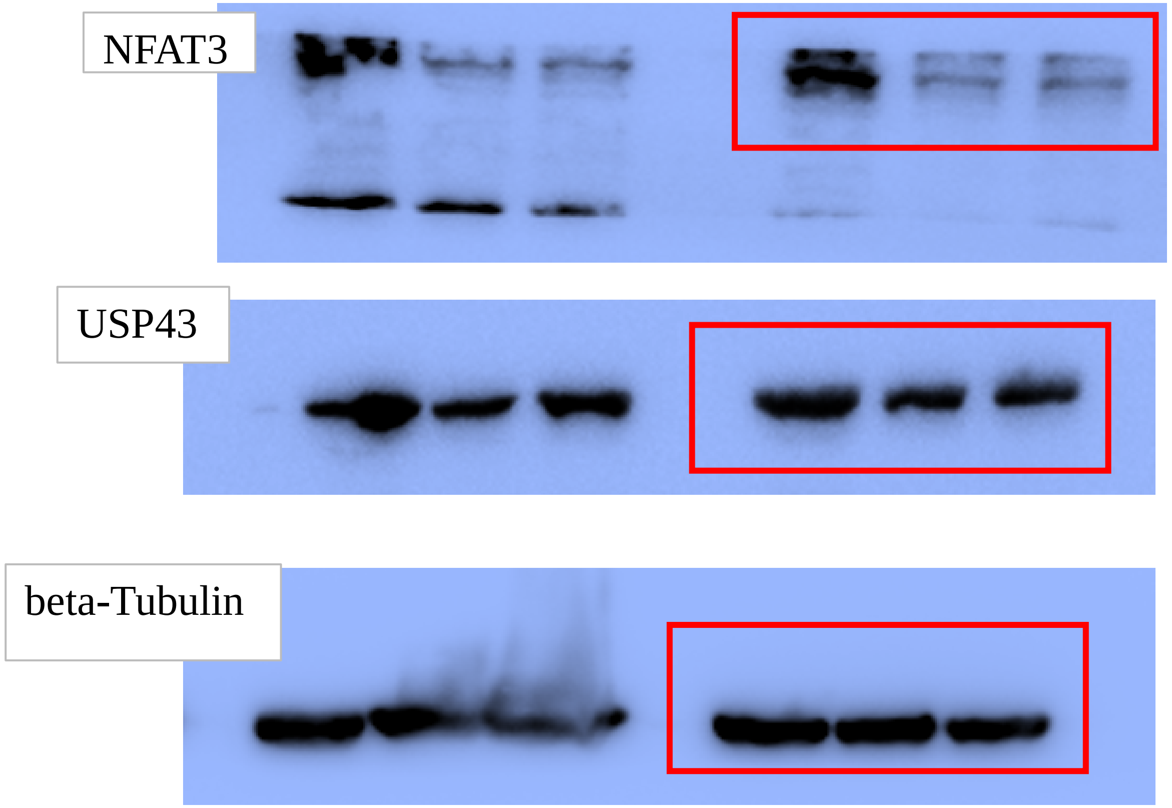

Supplement: Supplementary file 4 — Supplementary Figure [file 41419_2022_5174_MOESM4_ESM.pdf]
